# Challenges and Prospects of Low‐Temperature Rechargeable Batteries: Electrolytes, Interfaces, and Electrodes

**DOI:** 10.1002/advs.202410318

**Published:** 2024-10-22

**Authors:** Yaxuan Yang, Lingfei Zhao, Yiyang Zhang, Zhuo Yang, Wei‐Hong Lai, Yaru Liang, Shi‐Xue Dou, Min Liu, Yun‐Xiao Wang

**Affiliations:** ^1^ Key Laboratory of Advanced Functional Materials Ministry of Education School of Materials Science and Engineering Beijing University of Technology Beijing 100124 China; ^2^ Institute for Superconducting & Electronic Materials Australian Institute of Innovative Materials University of Wollongong Innovation Campus Squires Way North Wollongong NSW 2500 Australia; ^3^ School of Materials Science and Engineering Xiangtan University Xiangtan Hunan 411105 China; ^4^ Institute of Energy Materials Science University of Shanghai for Science and Technology Shanghai 200093 China

**Keywords:** aqueous electrolyte, battery, electrolyte/electrode interface, low temperature, solvation structure

## Abstract

Rechargeable batteries have been indispensable for various portable devices, electric vehicles, and energy storage stations. The operation of rechargeable batteries at low temperatures has been challenging due to increasing electrolyte viscosity and rising electrode resistance, which lead to sluggish ion transfer and large voltage hysteresis. Advanced electrolyte design and feasible electrode engineering to achieve desirable performance at low temperatures are crucial for the practical application of rechargeable batteries. Herein, the failure mechanism of the batteries at low temperature is discussed in detail from atomic perspectives, and deep insights on the solvent–solvent, solvent–ion, and ion–ion interactions in the electrolytes at low temperatures are provided. The evolution of electrode interfaces is discussed in detail. The electrochemical reactions of the electrodes at low temperatures are elucidated, and the approaches to accelerate the internal ion diffusion kinetics of the electrodes are highlighted. This review aims to deepen the understanding of the working mechanism of low‐temperature batteries at the atomic scale to shed light on the future development of low‐temperature rechargeable batteries.

## Graphical abstract

Low‐temperature performance of rechargeable batteries is crucial for their practical applications. This review comprehensively reveals the challenges and solutions for low‐temperature aqueous and non‐aqueous rechargeable batteries from an atomic perspective, deep insights on the solvent–solvent, solvent–ion, and ion–ion interactions in the electrolytes are provided, recent advances in the rational design of electrolytes, interfaces, and electrodes are included.

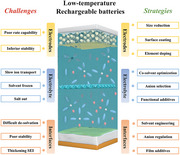

## 1 Introduction

**Figure 1:**
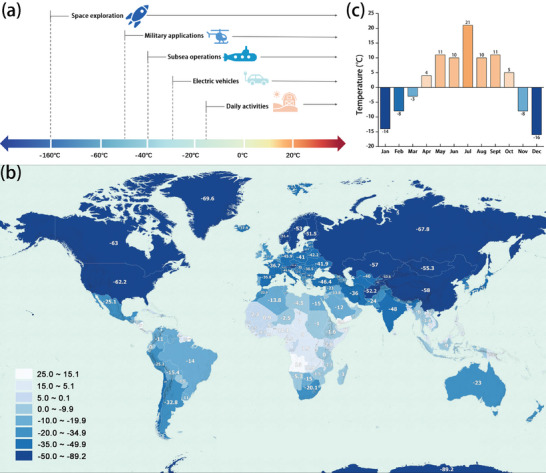
a) Various scenarios of human activities that require rechargeable batteries working at low temperatures. b) Map of the coldest recorded temperatures in various countries/continents of the world. Resources from https://vividmaps.com/. Reproduced with permission. Copyright 2024, Vivid Maps. c) The lowest monthly temperatures in Beijing in 2023. Data from China Weather Network.

With the ever‐increasing population and the impacts on the environment as well as the rapid decrease in natural resource reservations, the utilization of clean sources of energy, including wind, solar, wave, and tidal energies in nature have been considered feasible alternatives to address these problems.^[^ ^1^ ^]^ Rechargeable batteries are promising energy storage systems for the utilization of clean energy, which could store the energy generated from intermittent renewable energy sources.^[^ ^2^ ^]^ Rechargeable batteries working with metal ions in various valence states as charge carriers have been intensively studied, such as Li^+^, Na^+^, K^+^, Zn^2+^, Mg^2+^, Al^3+^, etc.^[^ ^3^ ^]^ To date, the research on rechargeable batteries has been mainly focused on room temperature, although a wide variety of human activities on earth and in space requires reliable rechargeable batteries with desirable all‐climate performance. Low temperature operation is vitally important for rechargeable batteries, since wide applications in electric vehicles, subsea operations, military applications, and space exploration are expected to require working at low temperatures ranging from 0 °C to as low as −160 °C (**Figure** **1**a).^[^ ^2^, ^4^ ^]^ On the other hand, the diversity of the Earth's climates results in low temperature records of 0 to −80 °C in a majority of landscapes (Figure 1b), and quite a lot of these areas exhibit a minimum temperature lower than 0 °C in several months of the year.^[^ ^5^ ^]^ For instance, in the year 2023, Beijing, China exhibited the daily minimum temperatures below 0 °C for over 5 months (Figure 1c).^[^ ^6^ ^]^ Consequently, there are many scenarios where the rechargeable batteries need to be working at low temperatures, which is essentially important for their practical applications.

The low temperature performance of rechargeable batteries, however, are far from satisfactory for practical applications. Serious problems generally occur, including decreasing reversible capacity and poor cycling performance.^[^ ^7^ ^]^ The degradation of the battery performance at low temperature could originate from the significant changes with temperature in electrolytes, interfaces, and electrodes. 1) First of all, the enhancement of the forces in the electrolyte caused by low temperature will lead to an increase in electrolyte viscosity, which could significantly reduce the speed of ion transportation. Further enhancement of the forces among solvent molecules at lower temperatures could lead to salt out and/or electrolyte freezing, which result in battery failures.^[^ ^8^ ^]^ 2) When the solvated ions reach the interfaces between the electrolyte and the electrodes, the strong ion–solvent interactions formed by polar solvents and ions at low temperature will make the de‐solvation difficult. Subsequently, a large number of solvated ions will accumulate on the surfaces of the electrodes, resulting in sluggish ion transportation kinetics.^[^ ^9^ ^]^ 3) The solid electrode interphase (SEI) formation is directly related to dynamic solvent removal. Low temperature would increase the interfacial resistance and limit ion transport, resulting in thick and uneven SEI layers, which hampers the cycling performance of the batteries.^[^ ^10^ ^]^ 4) Bare ion diffusion in the electrode is also a temperature‐dependent step. Even though the slower ion transport with decreasing temperature in the electrodes is not as severe as that in the electrolytes, it still reduces overall battery dynamics, thereby affecting the rate capability of the rechargeable batteries.^[^ ^11^ ^]^ In addition, the electrode structure is prone to change in the process of ion embedding and extrusion of the electrode, which causes the loss of electrode capacity. These limitations at low temperatures require optimization of both the electrolytes and the electrodes. In the case of the electrolyte, the solvation structure of an ideal electrolyte is not determined by a single interaction force, which depends on a balance of the solvent–solvent, solvent–ion, and ion–ion interactions in the electrolyte. The internal structure of the electrodes should also be rationally designed, since it impacts the ion transportation in the electrodes and therefore the overall battery performance at low temperatures.

Herein, this review focuses on these issues for low‐temperature batteries and provides a fundamental understanding of the changes in the physical and chemical properties of electrolytes and electrodes from an atomic perspective. The degradation mechanisms of the batteries at low temperatures are also discussed in detail. Furthermore, promising solutions through electrolyte, interface, and electrode optimization to achieve desirable low‐temperature performance are carefully elucidated. Moreover, research perspectives on future developments of low ‐temperature batteries have been provided. This review is expected to provide a deepened understanding of the working mechanisms of rechargeable batteries at low temperatures and pave the way for their development and diverse practical applications in the future.

## 2 Low‐Temperature Effects in Batteries

Low temperature will reduce the overall reaction rate of the battery and cause capacity decay. These failures of batteries at low temperatures are related to the obstruction of ion transport. The transportation of charge carriers in the discharge process of a battery involves ion extraction from the cathode material and across the cathode‐electrolyte interphase (CEI) and transfer of solvated ions through the bulk electrolyte, as well as the de‐solvation of the ions at the SEI and then their transport across the SEI and embedding into anode material. Under low temperatures, the viscosity of the electrolyte will increase, which results in sluggish ion transport, while further decreasing temperature would result in salt out and/or freezing of the electrolyte solvent. The de‐solvation of the ions at low temperatures will also be difficult due to the enhanced solvent–ion interactions, which affect the steady state of solid‐liquid two‐phase evolution.^[^ ^12^ ^]^ As for the electrodes, low temperature would slow down the ion diffusion and reaction rate in the electrodes. These issues concerning various components of the batteries will be discussed in detail in the subsequent sections.

### 2.1 Low‐Temperature Effects on The Electrolyte

**Figure 2:**
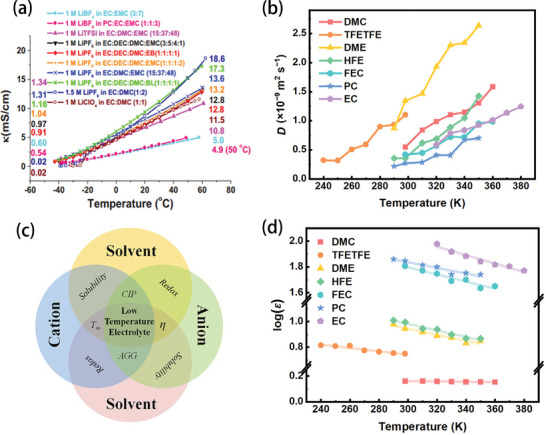
a) The temperature dependence of the specific conductivity for 10 electrolyte solutions. Conductivity values (in mS cm^−1^) measured at −40 °C and 60 °C for each solution are listed, respectively, on the left‐ and right‐hand sides of the graphs. Reproduced with permission.^[^ ^21^ ^]^ Copyright 2010, The Electrochemical Society. b) Variation of diffusion coefficients as the temperature increases. Reproduced with permission.^[^ ^13c^ ^]^ Copyright 2021, Wiley‐VCH GmbH. c) Schematic illustration of the interactions between various species in low‐temperature electrolytes. (*T*_m_, melting point; *η*, viscosity; CIP, contact ion pair; AGG, aggregates). Reproduced with permission.^[^ ^13b^ ^]^ Copyright 2023, Wiley‐VCH GmbH. d) Temperature dependence of the dielectric constants for various frequently used solvents. Reproduced with permission.^[^ ^13c^ ^]^ Copyright 2021, Wiley‐VCH GmbH.

The low‐temperature performance of an electrolyte is determined by the three types of forces inside the electrolyte: solvent–solvent, solvent–ion, and ion–ion interactions.^[^ ^13^ ^]^ These interactions significantly affect the constitution of the solvation sheath for the ions, which, in turn, is directly linked to the ionic conductivity of the electrolyte.^[^ ^14^ ^]^ As shown in **Figure** **2**a, the ionic conductivities of the electrolytes decrease with decreasing temperature, which is rationally expected as a result of increasing intermolecular interactions. The ionic diffusion coefficients for various solvents also exhibit a decreasing trend at lower temperatures (Figure 2b).^[^ ^13c^ ^]^ This is because as the temperature decreases, the dynamics of the interactions between the ions and molecules change accordingly. According to the “quadrilateral” interactions between ions and molecules at low temperatures, the interactions can be sorted into ion–ion, ion–dipole, and dipole–dipole interactions (Figure 2c).^[^ ^13b^ ^]^ The potential energies (*U*) associated with the three forces can be determined using the following equations.

(1) $$U_{\mathrm{ion}-\mathrm{ion}}=-\frac{1}{4\pi \varepsilon }\frac{z_{1\;}z_{2}e^{2}}{r}$$

(2) $$U_{\mathrm{ion}-\mathrm{dipole}}=-\frac{1}{4\pi \varepsilon }\frac{ze\mu cos\theta }{r^{2}}$$

(3) $$U_{\mathrm{dipole}-\mathrm{dipole}}=-\frac{1}{{\left(4\pi \varepsilon \right)}^{2}}\frac{2\mu_{1}^{2}\mu_{2}^{2}}{3k_{B}Tr^{6}}$$

where *ε, ze, µ, r, θ, k_B_*, and *T* represent the dielectric constant, the charge of the ion, the dipole moment of the dipole, the distance between ions or the centers of dipoles, the angle of the dipole relative to the line connecting the ion and the center of the dipole, the Boltzmann constant, and the absolute temperature, respectively.^[^ ^13^, ^14^ ^]^

According to the equations, as the temperature decreases, intermolecular thermal motion diminishes, leading to reduced spacing between molecules and an increase in ion–ion and ion–dipole interactions. Additionally, the dipole–dipole interaction is more sensitive to changes in intermolecular distance and temperature, and thus increases more rapidly as the temperature decreases.^[^ ^15^ ^]^ This changes the conventional focus on the ion–solvent interactions, while ignoring the solvent–solvent interactions in the study of room temperature batteries, due to the small van der Waals forces or scarce hydrogen bonds (HBs) among the solvent molecules.^[^ ^16^ ^]^ The HB is also a typical form of dipole–dipole interaction. It is well known that water as the solvent in an aqueous electrolyte will form a fixed HB network at low temperatures when the HB energy is greater than the kinetic energy of the water molecules, which is responsible for the freezing of water at low temperatures.^[^ ^12^, ^13^, ^14^, ^15^, ^17^ ^]^

The dielectric constant is generally used to represent the polarity of the solvent, and the relationship between the dielectric constant and the temperature is expressed by Equation (4).

(4) $$\varepsilon \;\left(T\right)=Ae^{\frac{E^{*}}{k_{B}T}}$$

where *A* and 𝐸∗ are parameters. The parameter 𝐸∗ is defined as the energy barrier to overcome the intermolecular interaction, which can represent the sensitivity of the dielectric constant of the solvent to change with temperature. This also illuminates the point that the interaction between dipoles affects the dielectric constant, which is generally used to measure the physical and chemical properties of solvents, such as their viscosity, salt solubility in electrolytes, etc. A solvent with a high dielectric constant has great polarity and strong solubility towards the salt solute.^[^ ^13^, ^18^ ^]^ With decreasing temperature, however, the barrier between molecules gets higher. The stronger interaction leads to an increase in the dielectric constant, and further strengthening of the force between molecules in the electrolyte will lead to slow kinetics in the bulk electrolyte, including decreased ionic conductivity and salt precipitation (Figure 2d).^[^ ^13c^ ^]^

To alleviate the high dielectric constant at low temperatures, a solvent with a low dielectric constant can be added to effectively adjust the overall dielectric constant of the electrolyte.^[^ ^16^, ^19^ ^]^ For example, Smart et al. found that low ethylene carbonate (EC) content is preferred at low temperatures because of its high dielectric constant (*ε* = 89.78) at room temperature, and they explored electrolytes containing EC with the addition of other co‐solvents, namely carbonate‐based quaternary low‐temperature electrolytes (1.0 M LiPF_6_, EC‐DEC‐DMC‐EMC, 1:1:1:3, where DEC is diethyl carbonate, DMC is dimethyl carbonate, and EMC is ethyl methyl carbonate) for lithium‐ion batteries (LIBs), which delivered 74.6% and 68.9% of the room temperature capacity at −40 and −50 °C, respectively.^[^ ^20^ ^]^

Selecting a solvent with a low dielectric constant alone is not a guaranteed solution, however, as it may lead to incompatibility with the solute salt. This is because ionic conductivity is influenced by the affinity between the organic solvent molecules and the ions.^[^ ^22^ ^]^ Generally, when the main solvent has a low dielectric constant, a polar solvent with a high dielectric constant can be chosen to assist in the salt dissolution, or a high polarity main solvent with an additive that has a low dielectric constant as another feasible alternative.

### 2.2 Low‐Temperature Effects on SEI Evolution

**Figure 3:**
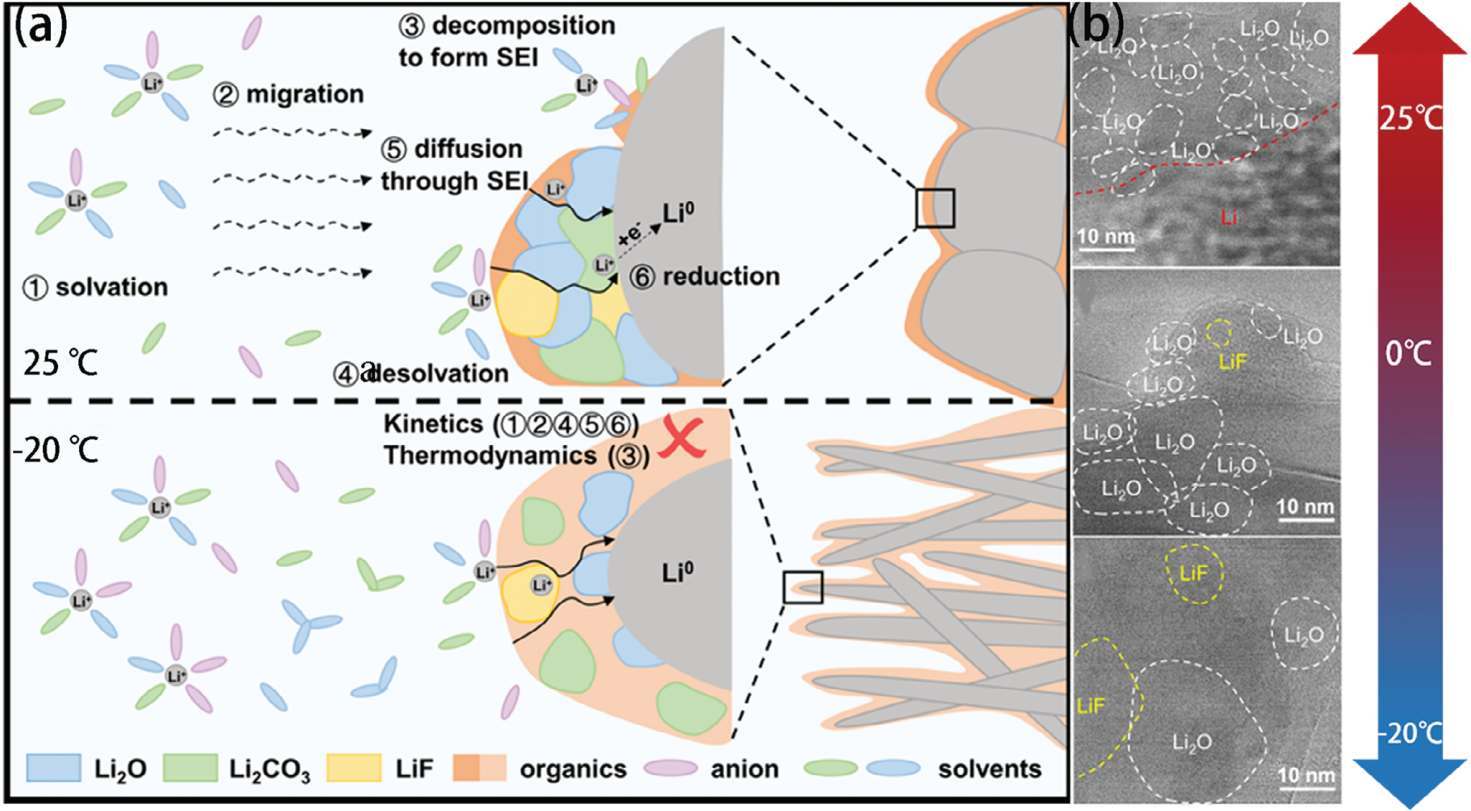
a) Schematic illustration comparing the ion diffusion and charge transfer at room temperature and low temperature for lithium plating. b) Cryogenic high‐resolution transmission electron microscope (cryo‐HRTEM) images of lithium metal deposited by 1 mol L^−1^ lithium bis(fluorosulfonyl)imide (LiFSI) in methyl trifluoroacetate: fluoroethylene carbonate (MTFA:FEC) (8:2) electrolyte at 25, 0, and −20 °C.^[^ ^26^ ^]^

The studies on the solvation structure of electrolytes and the interface model of de‐solvation have deepened our fundamental understanding of the generation and evolution of the SEI and CEI.^[^ ^23^ ^]^ When the ions arrive at the electrolyte‐electrode interface after being transported through bulk electrolytes, the first de‐solvation process with sluggish kinetics at low temperatures will become the major rate‐determining step.^[^ ^24^ ^]^ As can be inferred in Equation (2), the decrease in temperature would shorten the distance between the ion and the dipole and increase the interactions, leading to a maximum energy barrier of 50–70 kJ mol^−1^.^[^ ^9^ ^]^ Consequently, the decrease in temperature makes it harder for the de‐solvation of the ions to occur, resulting in accumulation of the solvated ions on the surface of the SEI, together with significant amount of non‐accessible capacity and a dramatically reduced reaction rate of the batteries. Besides, the decrease in temperature will lead to a change in the solvation structure of the electrolyte, which tends to form metastable SEI layers with more organic components. It will also lead to an uneven distribution of the components due to slow ion transport in the interphase and hamper the cycling performance of the battery.^[^ ^25^ ^]^ The impact of low temperature on the properties of the SEI layers has been revealed by several studies. For instance, the inorganic content in the SEI at 25 °C is more than that at −20 °C (**Figure** **3**a),^[^ ^25^ ^]^ which can also be seen from the cross‐section of the SEI formed on a Li metal anode (Figure 3b).^[^ ^26^ ^]^ Consequently, Li dendrites are prone to be generated on Li metal anodes at low temperatures. Previous studies demonstrated that when the temperature decreases, the interaction force of all the components in the electrolyte increases. The solvent–solvent intermolecular force is more sensitive to temperature, which leads to an increase in anion‐cationic binding. It has been frequently reported that the contact ion pair (CIP) structure formed by anions and cations could generate an inorganic‐rich SEI structure, which is conducive to higher ion transport.^[^ ^27^ ^]^ Therefore, the electrolytes that can maintain the CIP structure at low temperatures could sustain stable cycling of the electrodes due to the formation of a more stable SEI. Whereas, in the case of a large temperature drop, the combination of anion and cation will make the electrolyte salt out, which directly causes failure of the batteries. The rate of SEI formation also follows the Arrhenius equation, so low temperature also decreases the rate of SEI formation.

In summary, the de‐solvation and ion transport within the interfaces are complementary processes. The choosing of weak solvation solvents could weaken the solvent–ion interaction force and promote the de‐solvation of ions at the interface, while weak solvent–ion interaction will promote ion–ion interactions in some cases, making it easier for anions in the electrolyte to participate in the solvation sheath of the cations. As a result, inorganic‐rich SEI films with high ionic conductivity could be generated due to the preferential reduction of anions, which forms inorganic‐rich SEI films and accelerates the diffusion of ions in the SEI films.^[^ ^28^ ^]^ The latest research also suggests that the dynamic evolution of the electrolyte configuration at the Li/electrolyte interface essentially determines the structural composition of the SEI.^[^ ^12^ ^]^ Further research on the formation and evolution of the SEI layers at low temperatures would be desirable for the development of low‐temperature rechargeable batteries.

### 2.3 Low‐Temperature Effects on The Electrodes

For working rechargeable batteries, apart from the ion transport in the bulk electrolyte and across the interphases as discussed above, the ion diffusion in the electrodes is also an important part of the battery dynamics.^[^ ^29^ ^]^ At low temperatures, Li^+^ ions move more slowly in the electrode materials and SEI films than in the liquid electrolytes.^[^ ^16c^ ^]^ Low temperature has a serious limiting effect on the diffusion of bare ions in the electrode, and also on the reversible capacity as well as the rate capability of the batteries. First, the capacity of the electrode, as illustrated by Wang et al. in Equation (5), *K* represents the degree of reaction equilibrium, or in other words, the maximum theoretical capacity of charge and discharge. Therefore, the temperature changes the potential and capacity of the electrode in a thermodynamic manner (where *H* is the enthalpy, *K* is the equilibrium constant).^[^ ^30^ ^]^

(5) $$\frac{\partial lnK}{\partial T}=\frac{\Delta H}{RT^{2}}\;$$

Next, the kinetics of the electrode reaction mainly includes charge transfer and ion transport, and some studies have shown that at −20 °C, the charge transfer resistance (*R*_ct_) occupies almost 100% of the battery impedance. The Arrhenius equation also proves that the electrode resistance increases and the reaction rate decreases at low temperature. According to the Arrhenius equation, the rate coefficient *k* of a reaction can be determined using Equation (6) (where *A* represents the pre‐exponential factor and *E_a_* denotes the activation energy).

(6) $$k=Ae^{-E_{a}/RT}$$

**Figure 4:**
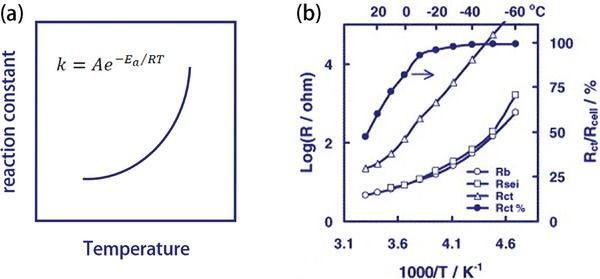
a) The relationship between temperature and the reaction constant. b) Temperature dependence of bulk resistance (*R*_b_), the resistance of the SEI (*R*_SEI_), and *R*_ct_, as well as the *R*_ct_ percentage. Reproduced with permission.^[^ ^11^ ^]^ Copyright 2003, Elsevier.

It can be inferred that the larger the *E_a_*, the more temperature‐sensitive is the reaction. A lower temperature would also lead to a lower reaction rate for the electrode (**Figure** **4**a), as the temperature drop limits the diffusion of ions inside the electrode. The relation between the ion diffusivity *D* and the operating temperature *T* can be empirically expressed as Equation (7) (where *D_0_* denotes the frequency factor, considered constant, *Q* represents the activation energy of diffusion, and *k_B_* is the Boltzmann constant).^[^ ^31^ ^]^

(7) $$D=D_{0}\;e^{-Q/k_{B}T}$$

It can be seen from Equation (7) that the temperature and activation energy affect the electrode at the same time, and the ion diffusion will decelerate when the temperature is lower. The inherent transport rate (*D*_Kion_) can also be expressed as Equation (8):^[^ ^10^, ^32^ ^]^

(8) $$D_{Kion}=-\frac{4kTm_{B}V_{m}I_{o}}{\pi c_{A}q^{2}z_{A}^{2}M_{B}S^{2}}{\left(\frac{\Delta E_{s}}{\Delta E_{t}}\right)}^{2}\;\left(t\ll \frac{L^{2}}{D_{ion}}\right)$$

where *m_B_*, *L*, and *S* are the mass, thickness, and surface area of the electrode, and *t* is the charge or discharge time. Δ*E_t_* represents the potential variation during a constant‐current pulse, while Δ*E_s_* reflects the steady‐state potential change observed between interruption steps. *M_B_* denotes the molecular weight, and *V_m_* represents the molar volume, *q* represents the elementary charge, *z_A_* denotes the charge number of species of *A*, *c_A_* represents the concentration of species of *A*. This formula also shows that temperature reduction will hinder diffusion and slow down the reaction kinetics in the batteries. On the other hand, it has been reported that at ‐20 °C, the *R*_ct_ occupies almost 100% of the battery impedance (Figure 4b).^[^ ^33^ ^]^ The dramatically increased charge transfer resistance would cause large voltage hysteresis and decrease the reversible capacity of the rechargeable batteries.

**Figure 5:**
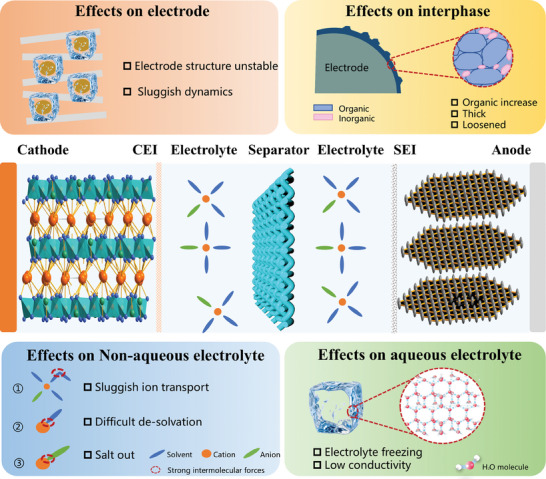
Effects of low temperatures on non‐aqueous electrolyte, aqueous electrolytes, interphases, and electrodes.

Therefore, low temperatures clearly have a significant impact on all aspects of battery operation, and as shown in **Figure** **5**, battery degradation is related to problems with electrolytes, electrodes, and interphases. For electrolytes, any significant increase in the intermolecular interaction force due to a decrease in temperature will limit the low temperature performance of the electrolyte. For example, the increase in the solvent–solvent interaction force will slow down the electrolyte flow and could even cause it to freeze. If the ion–ion interaction is too strong, salting out will occur and cause battery failure. Intensive solvent–ion interactions will make it extremely difficult to dissolve the electrolyte salt, and many solvated molecules reaching the surface of the interphase cannot be utilized, which tends to generate SEI layers with high organic content and non‐uniformity. In the case of electrode materials, in addition to the electrochemical changes inside the electrode and the decreased ion diffusion due to low temperature, the morphological structure of the electrode also changes at low temperature, which has a significant impact on the performance of the battery.^[^ ^34^ ^]^ The side reactions will become even more serious at low temperatures, and lithium dendrite growth in lithium metal batteries would be exacerbated at low temperatures.^[^ ^35^ ^]^ For the electrodes with problems even at room temperature, optimization of the electrolyte alone is not enough to change their performance at low temperatures, so further modifications of the electrode materials are required. Therefore, structural engineering, for example, by shortening the transmission path and changing the spatial structure, offers important methods to improve the low temperature performance of electrodes.

## 3 Electrolyte Engineering

**Figure 6:**
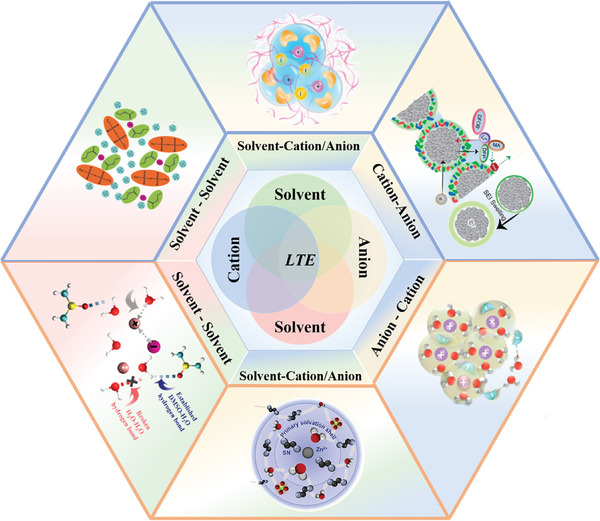
Optimization strategies for low‐temperature electrolytes. The top section illustrates the typical interactions in non‐aqueous electrolytes, the bottom section illustrates the typical interactions in aqueous electrolytes.

The properties of the electrolytes, especially the ionic conductivity of the electrolytes, are vitally important for the low temperature performance of batteries. Because aqueous and non‐aqueous batteries generally work with strikingly different working mechanisms, they will be discussed in different parts in the following sections. Regardless of whether an electrolyte is aqueous or non‐aqueous, failures at low temperatures are related to the changes in the interaction forces due to the influence of temperature. In non‐aqueous electrolytes, a significant decrease in the temperature makes the dipole–dipole, dipole‐ion, and ion–ion interactions in the electrolyte become very strong, which results in slow solution kinetics, difficult dissolution, and salting out, respectively. In contrast, if the three interaction forces are too weak, the electrolyte will show low ionic conductivity and the solute will precipitate easily, resulting in too many organic components in the SEI. It is crucial for an ideal electrolyte to coordinate and balance the interactions of the different components. In the case of aqueous electrolytes, the increase in HB interaction is the main culprit in low temperature freezing, so the current strategy has been to slow down the molecular interaction in water and break the HB, whether it is to weaken the dipole–dipole interaction, strengthen the dipole‐ion interaction, or enhance the ion–ion interaction formed by the water‐in‐salt (WIS) electrolyte structure.^[^ ^36^ ^]^ Too few HBs, however, will affect the stability of the electrochemical window, which indicates that the work on aqueous electrolyte still needs to consider the balance of these interactions (**Figure** **6**).

### 3.1 Non‐Aqueous Electrolytes

#### 3.1.1 Weakening Solvent–Solvent Interactions

**Figure 7:**
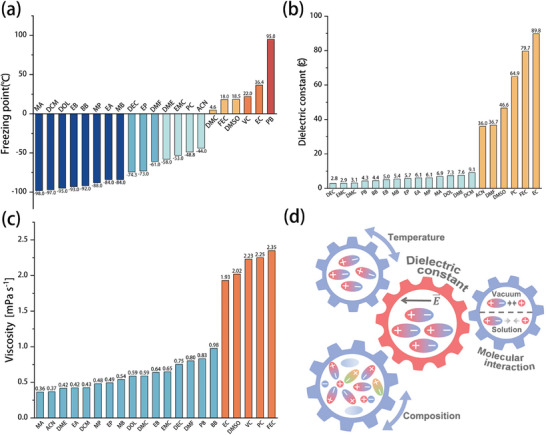
a) Freezing point, b) dielectric constant, and c) viscosity comparison of typical solvents for non‐aqueous electrolytes. d) Schematic representation of the relationships among temperature, electrolyte composition, dielectric constant, and molecular‐level interactions. Reproduced with permission.^[^ ^13c^ ^]^ Copyright 2021, Wiley‐VCH GmbH.

The electrochemical properties of electrolyte solvents, such as freezing point, dielectric constant, and viscosity, are crucial for the screening of non‐aqueous electrolytes. Accordingly, these properties are systematically summarized in **Figure** **7**. The dielectric constant is a measurement parameter expressing the polarity between solvents with temperature. A high dielectric constant indicates that the interaction force of the solvent molecules is large and that the solubility of the salt solute is high, which promotes the ion activity. As the temperature continues to decrease, however, the dielectric constant increases, the intermolecular force further increases, and the viscosity also increases, which is not in line with the goal of a high‐quality low‐temperature electrolyte. In the design of a low temperature electrolyte, the objectives of a low freezing point and low viscosity can ensure that the electrolyte does not freeze at low temperature and has good fluidity. It can be found from the statistical data that, although these parameters have no functional relationship, they also have certain outcomes, such as that the freezing point and viscosity of high dielectric constant solvent are relatively high. Therefore, at the beginning of the electrolyte design, these parameters can be simply screened, and big data can be used to design electrolytes that meet low temperature applications. And as discussed above, the difference in composition is also an important reason for affecting the dielectric constant.^[^ ^16^, ^19^ ^]^

In the case of the solvents with high dielectric constants which are not suitable for use as a single solvent at low temperatures, it is often necessary to use some co‐solvents to alleviate the problem of low temperature failure. As a common constituent of commercial electrolytes, the physical and chemical properties of EC render it unsuitable for batteries working in low‐temperature environments. The development of electrolytes with low content or even no EC is essentially necessary. The exploration of new electrolyte systems is also constantly impacting traditional electrolytes, although single‐solvent electrolytes still find it difficult to achieve excellent performance at low temperatures. New co‐solvents can take advantage of their low‐temperature performance in electrolyte solvents and achieve a win–win effect of high performance and economic benefits by mixing with traditional solvents.

The electrolyte composition, cathode, anode, and electrochemical performance of representative state‐of‐the‐art reports on non‐aqueous low‐temperature rechargeable batteries have been summarized in **Table** **1**. In previous studies, it was found that to obtain good performance at temperatures below ‐30 °C, the addition of ester solvents allows the EC content in the electrolyte to be reduced to less than 25% (volume ratio), and the best performance can be achieved at 15–20% EC content. Smart et al. also achieved low‐temperature operation of the electrolyte by adding ester co‐solvents, in a fixed ratio of 1.0 M LiPF_6_ in EC + EMC + methyl propionate (MP) (20:60:20 v/v %), which delivered over 6 times the capacity of a full carbonate blend and is capable of supporting moderate rates at low temperatures of −50 and −60 °C.^[^ ^37^ ^]^ Since some ester additives exhibit low freezing points, adding them to the electrolyte can also reduce the freezing point of the electrolyte.^[^ ^38^ ^]^

**Table 1:** Summary of state‐of‐the‐art reports on non‐aqueous low‐temperature rechargeable batteries.

Electrolyte	Electrodes	Operating temperature [°C]	Capacity [mAh g^−1^]	Rate [C]	Cycles	Reference
2 m LiTFSI, EA	PTPAn||PNTCDA	−70	18	5	NA	[39]
5 m LiTFSI, EA/DCM (1:4)	Li||PI	−70	60	0.2	100	[40]
1 m LiFSI, MB/PC (1:2)	Li||CF_x_	−70	240	0.1	N/A	[41]
1:10 LiPF_6_, PC/DEC (1:2), 5% FEC	Graphite ||LiNi_0.5_Mn_0.3_Co_0.2_O_2_	−40	430	0.1	N/A	[42]
1 m LiTFSI, DME/DOL (1:1), 1% LiNO_3_ + 50% AMDS	Li||S	−60	145	0.5 mA cm^−2^	20	[43]
1.2 m LiFSI, TEP/HFE (1:1)	Sb||LiFePO_4_	−20	103	0.1	N/A	[15]
1.87 g LiFSI, DME/FB (1.2:3)	Li||Se_0.05_S_0.95_@pPAN	−20	600	0.5	300	[44]
1.6 m LiFSI, FEC/DME/HFE (2:2:6)	Li||NCM523	−20	120	0.5	200	[45]
1.28 m LiFSI, FEC/FEMC/D2 (1:2:7)	Li||LiNi_0.8_Co_0.15_Al_0.05_O_2_	−85	96	N/A	N/A	[46]
1 m LiPF_6_, DFEC/DEC	Li|| LiNi_0.8_Co_0.1_Mn_0.1_O_2_	−30	93	0.2	50	[17a]
0.8 m NaOTF, THF/DME (3:1)	Na||NTP	−40	45.1	1	400	[47]
1.3 m LiFSI, DME/TFEE (2:8), 1 wt.% LiFMDFB + 0.05 wt.% AgNO_3_	Li||LiCoO_2_	−20	100	0.2	300	[48]
1 m LiPF_6_, FEC/AN (7:3)	Li||graphite	−20	360	0.3	N/A	[49]
LiFSI:AN:FB(1:2.4:3), 2% VC	Li||LTO	−40	100	0.1	200	[9a]
2 m LiTFSI, DEFM/FEC (8:2)	Li||LFP	−40	112.1	0.2	100	[50]
1.2 m LiTFSI, 1 m AN in FM	Li||NMC	−20	140	1/3	200	[51]
1 m LiDFOB, FEC/DMS/IF (4:7:9)	Li||LCO	−70	110	1/15	170	[52]
1.72 m LiFSI, DME/TTE (2:7)	Li||Ni_0.8_Co_0.1_Mn_0.1_O_2_	−20	150	0.2	5	[53]
0.8 m NaPF_6_, FEC/EMC/HFE (3:3:4)	Na||NVPOF	−30	90	0.5	600	[54]
0.8 m LiTFSI + 0.2 m LiODFB, ADN/EC (1:1)	Li||LTO	−20	100	1	100	[55]
0.5 m LiDFOB + 0.5 m LiBF_4_ + 0.05 m LiPF_6_, MA/DFEC (9:1)	Gr/SiO_x_||NCM811	−20	164.5	0.2	N/A	[56]
0.8 m NaOTf + 0.2 m NaBF_4_, DEGDME	Na||NVP	−40	50	0.2	80	[57]
0.3 m NaClO_4_, EC/PC (1:1), 5% FEC	Hard carbon||Na_3_V_2_(PO_4_)_2_F_3_	−25	100	1	1000	[58]
0.3 m NaPF_6_, DEGDME/THF (7:3)	Na||Na_3_V_2_(PO_4_)_3_	−20	80	0.5	250	[59]
1 m LiPF_6_, EC/EMC (1:2, wt.%)/0.5 wt.% DMS	Graphite ||LiNi_0.5_Co_0.2_Mn_0.3_O_2_	−20	20	0.2	15	[60]
0.8 m LiTFSI + 0.2 m LiNO_3_, DOL/DME 8:2, 10% FEC	Li||LFP	−60	75	1/20	10	[61]
1 m LiPF_6_, MA/EC/DEC/EMC (3:1:1:1), 1 wt.% TMSP, 1 wt.% PCS	Carbon||LiNi_0.5_Mn_1.5_O_4_	−5	101.7	0.3	200	[62]
1 m LiPF_6_, EC/DMC (1:1), 0.2% LiNO_3_, 2 wt.% FS,5% PBF	Li||LiFePO_4_	−40	80	0.5	100	[63]

PTPAn, polytriphenylamine; PNTCDA, naphthalenetetracarboxylicdianhydride (NTCDA)‐derived polyimide; PI, polyimide; PAN, polyacrylonitrile.

**Figure 8:**
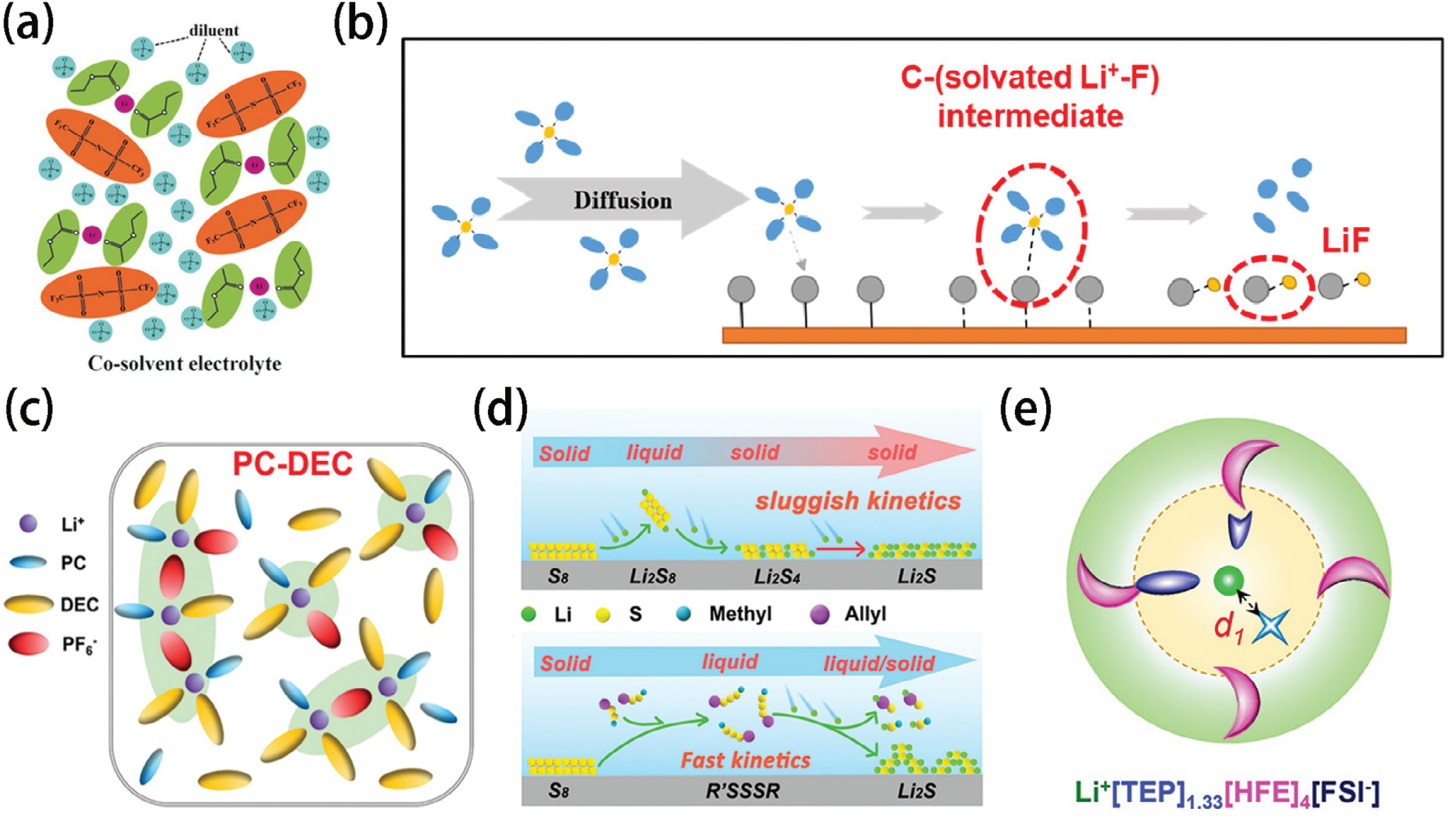
a) Solvation structure formed by DCM and solvent. Reproduced with permission.^[^ ^40^ ^]^ Copyright 2019, Wiley‐VCH GmbH. b) Illustration of the solvent‐involved discharge process in the Li||CF*_x_* cell; grey dots indicate F atoms, yellow dots represent Li^+^, orange plate the CF*_x_* electrode, and blue dots the solvent molecules. Reproduced with permission.^[^ ^41^ ^]^ Copyright 2021, Elsevier. c) Schematic illustration of the solvation structure of PC‐DEC electrolyte. Reproduced with permission.^[^ ^42^ ^]^ Copyright 2021, Wiley‐VCH GmbH. d) Schematic illustration of the phase conversion in Li–S batteries: in conventional electrolyte (top). In an allyl methyl disulfide (AMDS)‐modified electrolyte (bottom). Reproduced with permission.^[^ ^43^ ^]^ Copyright 2021, American Chemical Society. e) Solvation structure in 1.2 m LiFSI in TEP/HFE. Reproduced with permission.^[^ ^15^ ^]^ Copyright 2022, American Chemical Society.

Ethyl acetate (EA) exhibits a freezing point of −84 °C, which is significantly lower than those of most organic solvents typically employed in non‐aqueous electrolytes, rendering it a preferable component for low temperature electrolytes. The ionic conductivity of this electrolyte can reach 0.2 mS cm^−1^ at an ultra‐low temperature of −70 °C.^[^ ^39^ ^]^ To further improve the low‐temperature performance of electrolytes containing EA, the solvation structure is not destroyed, but low‐viscosity dichloromethane (DCM) is added around the high‐concentration salt (**Figure** **8**a). The results demonstrate that the 5 M lithium bis(trifluoromethanesulfonyl)imide (LiTFSI)/EA + DCM (1:4 by volume) electrolyte exhibits a low freezing point of −104.4 °C with a high ionic conductivity of 0.6 mS cm^−1^ at −70 °C.^[^ ^40^ ^]^

Propylene carbonate (PC) is also a commonly used electrolyte solvent. It has also been reported that, after exploration to uncover the cause of the temperature‐dependent change of Li^+^ coordination in electrolytes, it was found that a mixed solvent of PC: methyl butyrate (MB), 1:2 could break up the original solvation structure and allow more MB and bis(fluorosulfonyl)imide (FSI^−^) to participate in the Li^+^ solvation sheath, squeeze out PC molecules with high binding energy, and promote the dissolution of Li^+^ at low temperatures (Figure 8b). As a result, their lithium batteries delivered a high capacity retention of 28% at the ultra‐low temperature of −70 °C.^[^ ^41^ ^]^ It was demonstrated that increasing the Li^+^/PC molar ratio is an effective way to improve the electrochemical compatibility of the graphite anode in PC‐based electrolytes. When weakly coordinated DEC co‐solvent is introduced to promote the participation of PF_6_^−^ anions in the solvation sheath of Li^+^, anion‐induced ion–solvent‐coordinated (AI‐ISC) structures are formed (Figure 8c). This AI‐ISC structure results in a higher lowest unoccupied molecular orbital (LUMO) energy level for the electrolyte, significantly enhancing the reduction tolerance of PC solvents. Additionally, it ensures excellent low‐temperature performance at −40 °C when 5% fluoroethylene carbonate (FEC) is used as an additive.^[^ ^42^ ^]^

It has also been reported that adding solvated intermediates into the electrolyte could significantly enhance the reaction kinetics. For instance, Wang et al. successfully solved the problem of the slow solid‐solid conversion of Li_2_S_4_ and Li_2_S in conventional electrolytes by developing an all‐liquid reaction mechanism (Figure 8d). It enables stable cycling of Li–S batteries at −60 °C with an ultra‐high discharge capacity of 1563 mAh g^−1^.^[^ ^43^ ^]^ Similarly, a bifunctional co‐solvent, fluorobenzene (FB), has been introduced as an electrolyte additive for Li–S batteries, which enhances the solvation of dimethoxyethane (DME) and FSI^−^ around Li^+^ and inhibits the decomposition of DME. In addition, the reduction reaction of FB effectively increases the LiF content in SEI, which allowed the FB‐diluted highly concentrated electrolyte (DHCE) to maintain high electrical conductivity even at −20 °C.^[^ ^44^ ^]^ Recently, 1,1,2,2‐tetrafluoroethyl‐2,2,3,3‐tetrafluoropropyl ether (HFE) has been reported as a functional additive for electrolytes in LIBs, which could alter the solvation structure of the electrolyte and deliver enhanced low‐temperature performance. A novel nonflammable electrolyte, consisting of 1.2 m LiFSI dissolved in a mixture of triethyl phosphate (TEP) and HFE in a molar ratio of 1:3, was developed to stabilize the bulk alloying anode. A solvent–solvent, dipole–dipole interaction occurs between TEP and HFE, driven by the electronegative fluorine (δ^−^F) and electropositive hydrogen (δ^+^H), which not only attenuates the electrostatic interactions of Li^+^‐O_TEP_ (O atom of TEP molecule), but also compresses the solvated structure of Li due to the weak interactions of HFE with Li^+^, both of which result in enhancement of the FSI^−^/Li^+^ interactions. This is because the HFE‐TEP dipole–dipole interaction effectively reduces the TEP solvent and the polarization within the Li^+^ ‐TEP/HFE‐FSI^−^ complex (Figure 8e), thereby improving the stability (i.e., compatibility) of the electrolyte. Li‐ion batteries with the designed electrolyte can also be operated at low temperatures down to −20 °C. At a current density of 0.1 C, the capacity of 103 mAh g^−1^ can be maintained at 68.2% of the room temperature capacity.^[^ ^15^ ^]^

All these solvents are basically added to mitigate the failure of the main solvent at low temperatures, overcoming the slow ion transport or even freezing of the electrolyte due to the enhanced intermolecular interactions at lower temperature. Therefore, they are more suitable for dilute electrolytes in which the solvent is the main component. In the current bulk electrolyte, we still adopt the widely used cocktail strategy, by blending electrolytes with different dielectric constants to achieve our low temperature requirements. In the future, we should further organize the electrolyte database, and the technology combined with big data will advance our research and development of low‐temperature electrolytes. Future work should further explore co‐solvents and electrolyte mixtures that appropriately suppress ion aggregation while promoting the rapid migration of ions.

#### 3.1.2 Weakening Ion–Solvent Interactions

The solvation structure formed by various forces in the electrolyte is directly affected by the ambient temperature, which directly leads to the different reaction kinetics of (de)solvation. After ions are transported through bulk electrolytes, they begin to desolvate on the surfaces of the SEI/CEI and diffuse into it. The most energy‐intensive desolvation steps and the composition and structure of the SEI/CEI at low temperatures are directly related to the properties of the solvation sheath.^[^ ^9^ ^]^ Therefore, we need to construct a weak ion–solvent interaction, which, in addition to reducing the dissolution barrier of solvated carrier ions and achieving a faster desolvation process, can also promote the formation of CIP structures to produce a low‐temperature stable SEI. The weak solvent is an ideal low‐temperature electrolyte because it can produce only a weak force with ions.^[^ ^13b^ ^]^ First of all, the weakening of the ion–solvent interaction reduces the solvent‐removal barrier of solvated carrier ions, realizes a faster solvent removal process, and accelerates the reaction rate of the battery, which is of great significance for fast charging and low‐temperature operation. In addition, the weakening of the ion–solvent interaction is also the basis of the compatibility between graphite electrodes and electrolytes. For example, the rapid desolvation of Na^+^ on the electrode surface was achieved by the addition of the weak solvent tetrahydrofuran (THF), which was not achieved by PC electrolyte.^[^ ^64^ ^]^

**Figure 9:**
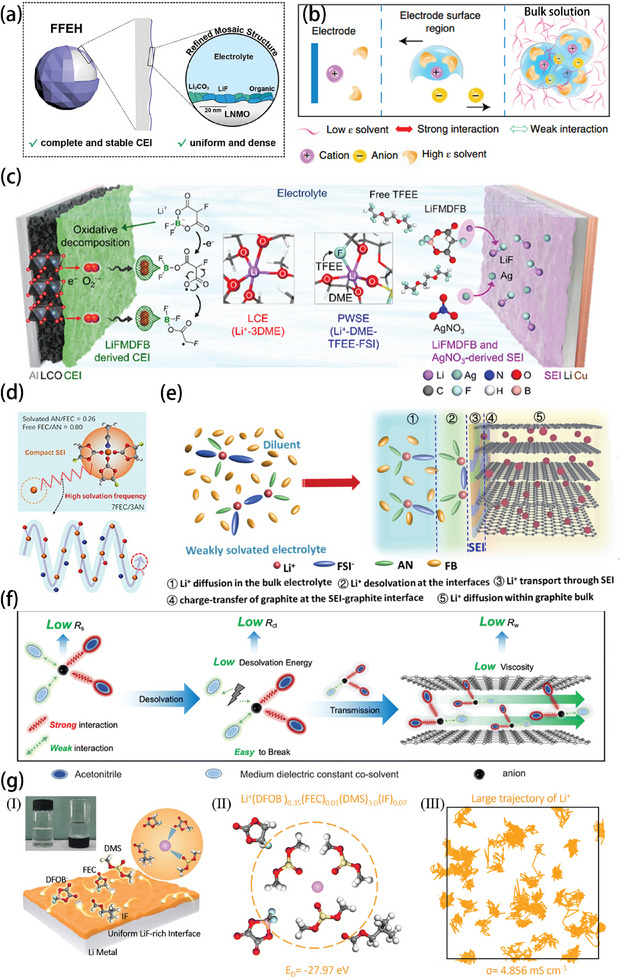
a) Schematic illustration of LiMn_1.5_Ni_0.5_O_4_ (LNMO) cathode particle configuration and CEI formation in FFEH electrolytes. Reproduced with permission.^[^ ^67b^ ^]^ Copyright 2024, Wiley‐VCH GmbH. b) The anticipated electrochemical processes occurring at the interface between the electrode and the electrolyte of the controlled electrolyte. Reproduced with permission.^[^ ^22b^ ^]^ Copyright 2019, Springer Nature. c) Proposed mechanisms for additive‐enhanced PWSE in a Li||LiCoO_2_ full cell. Reproduced with permission.^[^ ^48^ ^]^ Copyright 2023, RSC Publishing. d) Schematic illustration of the solvation structure and the solvation frequency in 7FEC/3AN electrolyte. Reproduced with permission.^[^ ^49^ ^]^ Copyright 2022, American Chemical Society. e) Schematic representations of the solution structures and the Li^+^ intercalation process within the graphite layers in AN‐DHCE. Reproduced with permission.^[^ ^9a^ ^]^ Copyright 2022, Elsevier. f) Schematic illustration of the binary solvent concept. Reproduced with permission.^[^ ^74^ ^]^ Copyright 2023, Royal Society of Chemistry. g) Schematic illustration of uniform Li rich interface and 45% IF electrolyte. (II) The Li^+^ solvation structures and corresponding desolvation energies of 45% IF electrolyte. (III) The migration pathway identified from the topology analysis of 45% IF electrolyte. Reproduced with permission.^[^ ^75^ ^]^ Copyright 2023, RSC Publishing.

In addition to accelerating the desolvation rate, weak solvent–ion interaction is conducive to circulation at low temperatures, and the ion transport rate inside the interface is also important for the rate performance of low‐temperature batteries. Vacancies and pores are considered to be the two main transport pathways of ions within the SEI.^[^ ^65^ ^]^ Functioning as a kind of vacancy, grain boundaries exist between multiple inorganic substances, which can greatly increase the ion carrier concentration and create a channel for Li^+^ migration.^[^ ^66^ ^]^ The tetrahedrally coordinated Li^+^‐(FEC)(DME)(FSI^−^)_2_ solvated clusters formed upon the addition of 1.6 m LiFSI to a mixed solvent of FEC/DME/HFE (referred to as 1.6‐FDH) are quite stable, and more LiF, Li_2_CO_3_, and Li_2_O inorganic products are produced by electrolyte reduction due to the introduction of anions. The contact between the multiple inorganic species promotes the accumulation of interfacial space charge, which lowers the Li^+^ transport barriers to synergistically increase the ionic concentration and accelerate the Li^+^ conduction. an Li||LFP battery, where LFP stands for LiFePO_4_, can achieve more than 100 reversible cycles at the low temperatures of −20 °C and the current density of 0.5 C.^[^ ^45^ ^]^ Also, by using HFE as a diluent, the Fan's team promoted the interaction between Li^+^ and PF_6_^−^ by HFE, and the resulting structures such as aggregates (AGG) and CIP were reduced to a dense and well‐passivated CEI layer of LiF/Li_2_CO_3_ at low temperatures, thus making the Li^+^ flux evenly distributed on the CEI interface (**Figure** **9**a). The FFEH (FEC/FEMC/EP/HFE) had a high Li^+^ conductivity (3.64 mS cm^−1^) at −20 °C.^[^ ^67^ ^]^ This indicates that the inorganic component of the SEI is conducive to ion transport, and the formation of the SEI is related to the reduction of anions, indicating that ion–ion interaction is an important factor in regulating SEI layers.^[^ ^12^ ^]^ Zhang's team also proposed that stable SEI and CEI layers can be generated at the same time through anion adjustment, and their graphite||LFP battery composed of LiFSI/MF91 delivered an ultra‐wide operating temperature range of −80 to 80 °C. It also had an impressive 1200‐cycle cycling performance at 2 C, a capacity retention rate of 80.2%, and extremely fast charging capability.^[^ ^68^ ^]^ The anions reversibly self‐assemble into a dense and ordered molecular layer on the CEI, which also contributes to the desolvation of the solvent.^[^ ^69^ ^]^

Fluorination of electrolyte solvents is a feasible approach to generate weak solvents. Incorporation of electron‐withdrawing fluorine groups in the solvent molecules could result in a lower lowest unoccupied molecular orbital (LUMO) and/or higher highest occupied molecular orbital (HOMO) for the solvent molecules, therefore reducing the energy gap between the LUMO and the HOMO. The preferential decomposition of the F‐containing solvents or additives could generate inorganic salt‐rich thin and stable SEI layers on the anodes, leading to increased oxidative stability and cycling stability of the batteries.^[^ ^70^ ^]^ This reduction enhances oxidation resistance and facilitates the formation of more stable CEIs and SEIs at both the cathode and anode interfaces.^[^ ^46^, ^66^, ^71^ ^]^ Fluorination can also be used to reduce the freezing temperature of EC‐based electrolytes and the affinity of EC with Li^+^ at low temperatures. For instance, when dissolving a fully fluorinated electrolyte in a highly fluorinated non‐polar solvent, the solvated Li^+^ ions will be separated from the anions in the surface region due to the influence of the electric field (Figure 9b). When Li^+^ reaches the electrode surface, the fluorocarbonate molecules will eventually break down. This makes the lithium‐ion transport process faster, and an Li||LiNi_0.8_Co_0.15_Al_0.05_O_2_ (Li||NCA) battery was able to operate in fluorinated 1.28 m LiFSI‐FEC/FEMC electrolyte, where FEMC stands for methyl (2,2,2‐trifluoroethyl) carbonate. This battery could still be used at −85 °C, providing 56% of its room temperature capacity.^[^ ^46^ ^]^ Wang et al. also accelerated the evolution of the Li^+^ solvation sheath by adjusting the degree of fluorination of the solvent, and the ion–dipole interaction strength decreased as the degree of fluorination increased. At −20 °C, ethyl difluorocarbonate (DFEC)‐based electrolytes delivered an enhanced ion dissolution rate that was six times faster than for non‐fluorinated EC‐based electrolytes. Li||LiNi_0.8_Co_0.1_Mn_0.1_O_2_ batteries based on DFEC electrolyte could still maintain 91% of the original capacity after 300 cycles at 25 °C and retained 51% of room temperature capacity at −30 °C. During this SEI formation process, the isolated DFEC in the solvated sheath was preferentially reduced to form a Li^+^ conductive SEI layer, resulting in uniform lithium deposition, which is also the reason for the good performance of lithium batteries.^[^ ^17^, ^72^ ^]^ It has also been reported that the addition of fluoroacetonitrile (FAN) could not only weaken the interactions between the ions and the solvent molecules, but also allow the anions to enter the first Li^+^ solvation sheath and form an inorganic‐rich interphase. Meanwhile, the solvent in the secondary solvation sheath can also pull the ions out to form a fast transport ligand channel. As a result, the electrolyte composed of 1.3 m LiFSI in FAN exhibits ultrahigh ionic conductivity of 11.9 mS cm^−1 ^at an ultralow temperature of −70 °C.^[^ ^73^ ^]^

In the case of sodium cells, the introduction of weak solvents could also solve the desolvation problem well. By incorporating the weak Na^+^ solvation co‐solvent tetrahydrofuran/1,2‐dimethoxyethane (THF/DME), the kinetic barrier for Na^+^ de‐solvation was notably reduced. In addition, the increase in anion–cation coordination generated an anion‐derived NaF‐rich SEI film on the sodium electrode, which inhibited the growth of dendrites and ensured the stable cycling of the battery at −60 °C.^[^ ^47^ ^]^ Similarly, the intervention of the weak solvent could also enhance the cycling performance of Li metal batteries. For instance, the introduction of 2,2,2‐trifluoroethyl 2‐fluoroethyl ether (TFEE) could alter the original solvent structure of Li‐DME into a partially weakly solvated structure bound to anions, optimizing the ionic desolvation kinetics and strengthening the resistance to capacity decay, while the ionic additives lithium fluoromalonato(difluoro)borate (LiFMDFB) and AgNO_3_ increase the electrolyte‐phase interfacial stability, and the PWSE partially and weakly solvating electrolyte (PWSE) system combined with the electrolyte additives was capable of operating over a wide temperature range from −20 to 60 °C. The working mechanism is shown in Figure 9c.^[^ ^48^ ^]^

Although weakly solvated electrolytes can accelerate the desolvation process and facilitate the formation of a stable interphase, weakly solvated molecules inevitably slow down the rate of ion transfer in the bulk electrolytes.^[^ ^76^ ^]^ Therefore, the jumping mechanism for ion transport caused by the addition of acetonitrile (ACN) results in a maximum increase in the solvation frequency of the 7FEC/3ACN electrolyte (Figure 9d). The solvation frequency is defined as the reciprocal of the residence time of solvents (FEC and ACN) within the Li^+^ cation solvation shell. A higher solvation frequency indicates that Li^+^ transitions more rapidly between different ligand configurations across a broad temperature range from −20 to 60 °C. This fast‐charging technology opens up new paths for low‐temperature rechargeable batteries.^[^ ^49^ ^]^ To further balance the poor Li^+^ transport of weakly solvated electrolytes in the bulk electrolyte, Sheng et al added acetonitrile (ACN) to an electrolyte containing fluorobenzene (FB), because the low binding energy of Li^+^ with ACN molecules and its high dielectric constant can enable it to better dissolve lithium salts and also to be easily separated from the solvation sheath (Figure 9e).^[^ ^9a^ ^]^ Dilute electrolytes can enhance the Li^+^ desolvation kinetics, thus improving the rate performance of low‐temperature lithium‐ion batteries.^[^ ^77^ ^]^ For instance, when 2% vinylene carbonate (VC) is added to AN‐DHCE, graphite||NCM811 (LiNi_0.8_Co_0.1_Mn_0.1_O_2_) pouch cells, demonstrated excellent rate performance. Even at temperatures as low as −40 °C, the lithium titanate anode retained 91.1% of its initial capacity after over 200 cycles. Also, by tailoring the electrolyte, the bonding strength in the solvated structure of the Li^+^ was weakened in the high‐concentration fluorinated carboxylate electrolyte, which facilitated the process of low‐temperature desolvation.^[^ ^50^ ^]^

Generally, a single solvation cannot cope with the transport in the two different stages of the bulk electrolyte and the dissolution process, and adapting to the transport needs of different stages is the direction of future research. Recently, Yang also considered the solvation requirements for the different charge transport stages and designed a binary solvent with a high dielectric constant solvent and a weak solvent, which can dissociate the solute salts to facilitate transport in the bulk electrolyte while lowering the de‐solvation energy (Figure 9f). *N*,*N*‐diethyl‐*N*‐methylethanaminium tetrafluoroborate (TEMA‐BF_4_) in ACN/acetone (ACT) electrolyte shows the highest conductivity, even at very low temperatures, demonstrating the superiority of the strong‐weak binary solvent structure for the ionic dynamics. This is an example of taking advantage of strong‐weak binary solvents to achieve a balance between conductivity and de‐solvation.^[^ ^74^ ^]^ Also, similar to the strong and weak binary solvents is the mixture of AN and fluoromethane (FM). In this electrolyte again, the strong binding of AN to Li^+^ makes almost all AN and Li^+^ completely solvated. The slight solvation of FM with Li^+^ provides an opportunity for rapid exchange and de‐solvation, which allows for high salt (1.2 M LiTFSI) and co‐solvent concentrations (1 M ACN in FM), and the liquefied gas electrolyte enabled stable cycling of an Li||LiNi_0.6_Mn_0.2_Co_0.2_O_2_ (NCM622) cell at the low temperature of −60 °C with a 45% capacity retention as compared to its room‐temperature (RT) capacity.^[^ ^51^ ^]^ Liu et al. designed electrolytes that also lower the desolvation energy to ‐27.97 eV, in which the Li^+^ solvation shell contains both difluoro(oxalato) borate (DFOB‐) and dimethyl sulfite (DMS)/molecules, and the FEC and isobutyl formate (IF) molecules hardly coordinate with Li^+^ (Figure 9g). This results in a distinctive electrolyte structure characterized by low solvation, high desolvation energy, elevated Li^+^ conductivity, and a large diffusion coefficient. The electrolyte exhibits a broad operational temperature range, extending from −70 to 60 °C.^[^ ^52^ ^]^

In summary, the ionic coordination environment or the solvation structure of the electrolyte, which involve ion–solvent and cation‐anion interactions, is critical for the low‐temperature operation of rechargeable batteries. It is worth noting, however, that when the solvation capacity of the weak solvent is too low, it is difficult to dissolve the solute, and the transport in the bulk electrolyte is slow. Properly coordinated solvents can improve performance at low temperatures, indicating that a thorough understanding and precise formulation of the solvation structure along with a careful balance of ion–solvent and ion–ion interactions during both solvation and desolvation are crucial for designing electrolytes that are effective for both rapid charging and low‐temperature applications.^[^ ^78^ ^]^

#### 3.1.3 Strengthening Ion–Ion Interactions

For high‐concentration electrolytes, unlike the many solvents mentioned above, the introduction of salt makes the interactions in the electrolyte more complicated due to the formation of salt‐binding and solvation structures. For the adjustment of the solvent structure, on the one hand, the addition of a weak solvent can weaken the ion–solvent interaction, so as to achieve strong ion–ion binding, thus forming an anion‐derived interface. For the adjustment of the solvent structure, we can also choose the right anion to realize the weak solvent structure of the electrolyte. The solvation behavior of a salt is regulated by the ion–solvent and cation‐anion interactions, which compete with each other in the electrolyte, and while too large an ion‐binding energy may result in low‐temperature salting out to a certain extent, suitable strong ion–ion binding can achieve the formation of an anion‐derived interphase and accelerate the ionic transport, so balancing the interacting forces of the substances in the electrolyte is of particular importance.

**Figure 10:**
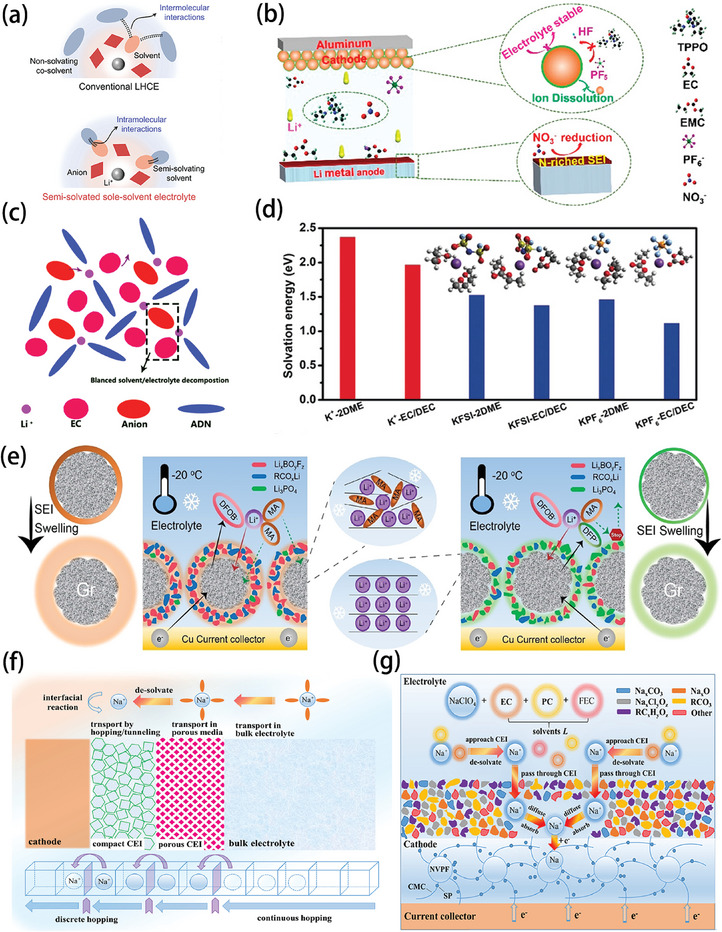
a) Schematic illustration of the solvation structures in the C‐LHCE and quasi‐solid electrolyte (QSE). Reproduced with permission.^[^ ^53^ ^]^ Copyright 2023, American Chemical Society. b) Schematic illustration of the interfacial chemistry at the interfaces. Reproduced with permission.^[^ ^80^ ^]^ Copyright 2021, American Chemical Society. c) The solvation structure between solvent and electrolyte is controlled by ADN. Reproduced with permission.^[^ ^55^ ^]^ Copyright 2021, Royal Society of Chemistry. d) Solvation energies of various salt‐solvent complexes with K^+^. Reproduced with permission.^[^ ^82^ ^]^ Copyright 2019, Wiley‐VCH GmbH. Schematic illustrations depicting the SEI swelling behavior and solvent co‐intercalation mechanisms in the graphite (Gr) anode for e) pristine and LiDFP‐modified systems at −20 °C. Reproduced with permission.^[^ ^56^ ^]^ Copyright 2023, Wiley‐VCH GmbH. f) Schematic diagram illustrating the reaction pathway of a typical electrode process along with the Na^+^ transport behavior. Reproduced with permission.^[^ ^58^ ^]^ Copyright 2021, Elsevier. g) A schematic diagram of the Na_3_V_2_(PO_4_)_2_F_3_ (NVPF)/interphase/electrolyte system in a weakly‐solvating electrolyte. Reproduced with permission.^[^ ^58^ ^]^ Copyright 2021, Elsevier.

The first consideration is the selection of the solvent. The addition of a weak solvent can enhance the ion–ion interaction because the weak solvent makes the interaction between solvent and cations in the solvation sheath weak, and then the anions can be inserted into the solvation sheath, thus forming strong ion–ion structures such as CIPs. Holoubek et al. also showed that the cells retained 84% and 76% of their room temperature capacity when cycled at −40 °C and −60 °C, respectively, due to a CIP structure consisting of diethyl ether (DEE) and LiFSI in a full cell with a sulfurized polyacrylonitrile (SPAN) cathode paired with a single excess Li metal anode, and their observations further confirmed the advantages of the CIP structure in achieving satisfactory low‐temperature performance of the Li metal anode. The charge transfer resistance is dominated by the desolvation of Li^+^ at ultra‐low temperatures. The weakly bound DEE system provides uniform deposition behavior at these ultra‐low temperatures, increasing the stability of the SEI, while 1,3‐dioxolane (DOL)/DME with high binding energy takes a dendritic approach to deposition under harsh conditions, increasing the cell resistance and shortening cell life.^[^ ^79^ ^]^ If the electrolyte decomposition caused by the unstable coordination of lithium in the DME‐based localized high‐concentration electrolyte (LHCE) is changed, the solvation structure can be stabilized by concentrating the solvated and non‐solvated fragments in the semi‐separated single‐solvent electrolyte into a single molecule (**Figure** **10**a), and at the same time, increasing the AGG content in the solution. Li||NCM811 batteries with this new electrolyte could be operated stably over a wide range of temperatures from −20 to 60 °C.^[^ ^53^ ^]^ For ions that promote diffusion in the SEI, Zheng et al. took a two‐pronged approach that was used to break the barrier of Na^+^ de‐solvation and SEI optimization through fluorination. The rapid diffusion of interfacial Na^+^ was achieved by a Na_15_Sn_4_/NaF biphasic artificial SEI. The battery had good low‐temperature performance.^[^ ^54^ ^]^

Some solvents can be selected as solubilizers while introducing anions into the first solvation sheath. Xiao et al. incorporated a high‐donor number (DN) multifunctional solvent, tris(pyrrolidinophosphine) oxide (TPPO) into a carbonate electrolyte. Due to its high affinity for Li^+^, TPPO causes LiNO_3_ to easily dissolve into such a carbonate electrolyte. Its structure is shown in Figure 10b. In the TPPO electrolyte, TPPO and NO_3_^−^ are effectively incorporated into the initial solvation shell of Li^+^. This leads to the preferential reduction of NO_3_^−^ at the anode‐electrolyte interface and the oxidation of TPPO at the cathode‐electrolyte interface. This results in the formation of a strong and highly ion conductive Li_3_N‐rich SEI film at the anode‐electrolyte interface, and a tough and thin N, P‐rich CEI film at the cathode‐electrolyte interface. Their cells exhibited excellent cycling stability, even over a wide operating temperature range of −15 to 70 °C.^[^ ^80^ ^]^ Anions can also be introduced by adiponitrile (ADN), which controls the competitive decomposition process between EC and the lithium salt, changing it from the original ion pairs separated by solvent (SSIP) structure of a single solvent to the CIP structure involving anions (Figure 10c), which increases the content of LiF in the CEI. Lithium metal batteries with LFP, lithium titanium oxide (LTO), and LiNi_0.5_Co_0.2_Mn_0.3_O_2_ (NCM523) as cathodes show excellent electrochemical performance in the temperature range of −20 to 150 °C.^[^ ^55^ ^]^

Second, in addition to solvent screening, anion selection can also control anion‐cation interactions, directly enhancing interactions in structures such as CIPs. There are signs of this practice, for instance, where the relatively large ionic radius and low Lewis acidity of K^+^ ions result in weaker interactions with the electrolyte and reduced solubility in non‐protonic solvents. However, potassium bis(fluorosulfonyl)imide (KFSI) can be well solubilized in mixed EC/DEC solvents, and at the same concentration, KFSI can be solubilized more easily than KPF_6_ with DME solvent molecules.^[^ ^81^ ^]^ The solvation energies of the KFSI‐based electrolytes are higher than those of electrolytes associated with KPF_6_, leading to a higher degree of solvation of the molecules, whereas the solvation energies of KFSI‐EC/DEC and KFSI‐2DME are lower than those of K^+^ with solvent alone, suggesting that the salt‐solvent interactions are weaker, favoring the diffusion and desolvation of the K^+^ ions (Figure 10d). In this regard, in addition to choosing a suitable solvent, anion blending can also control the dissolution of the salt, because different anions lead to different solvation energies.^[^ ^82^ ^]^ In addition to such lithium salts as LiBF_4_, which is the least dissociated among all Li salts, the strong electrostatic attraction between Li^+^ and BF_4_^−^ builds a weakly solvated electrolyte, which leads to a greater distribution of anions in the first solvation sheath, significantly reducing the number of SSIPs and improving the ratio of CIP/AGG in the electrolyte, which leads to better participation of anions in the formation of the cathode and anode electrolyte interphases, so that the low temperature stability of the battery is improved.^[^ ^31^, ^83^ ^]^

The strong binding of anions and cations can change the configuration of the solvated sheath. When the inorganic anion diisopropyl fluorophosphate (DFP^−^), which binds more strongly to lithium ions, was added to the solution, the primary solvation sheath (PSS) structure was remodeled, inhibiting the SEI swelling caused by the original organic anion, as shown in Figure 10e. The preferential participation of the inorganic anion generated a Li_3_PO_4_‐rich SEI without altering the original weakly solvating electrolyte (WSE) structure, and the LiDFP‐modified electrolyte system could reach 164.5 mAh g^−1^ initial capacity at −20 °C.^[^ ^56^ ^]^ Also used are the double salts sodium trifluoromethanesulfonate (NaOTf) and NaBF_4_, which act synergistically in diglyme solvent and lead to highly efficient sodium deposition/stripping down to −50 °C. Incorporation of NaOTf alleviates the problem of low conductivity due to the strong anionic binding of NaBF_4_. The high Coulombic efficiency achieved for sodium cycling surpasses previous findings with lithium metal, indicating that sodium metal holds significant potential for low‐temperature battery applications.^[^ ^57^ ^]^ For strongly bound lithium salts, such as lithium trifluoroacetate (LiTFA), it is possible to modulate the interdependent electrostatic interactions that occur within the electrolyte to prevent polysulfide aggregation from occurring. This leads to a significant increase in the conversion of Li_2_S_4_ to Li_2_S at low temperatures, since the strongly bound lithium salts disrupt the Li^+^‐S_4_ polymerization network to good advantage at low temperatures.^[^ ^84^ ^]^ This further confirms that, if one wants to get good battery performance, one needs to explore how to reduce the barriers to battery operation.^[^ ^42^ ^]^

Not only can the intermolecular forces be adjusted through the control of solute species, but changes in salt concentration can also change the solvation structure and adjust the intermolecular forces. The activation energy barrier for Na^+^ passing through NaF is greater than that for Na_2_CO_3_. Consequently, since inorganic components such as Na_2_CO_3_ and NaF primarily influence ionic conductivity, an ultra‐thin, dense inorganic SEI with a lower NaF content may facilitate better ion transport within the electrode.^[^ ^85^ ^]^ The transport of Na is shown in Figure 10f, where it is shown that the electrolyte with a low salt concentration of 0.3 m NaClO_4_ tends to form a weak SSIP solvent structure of Na^+^ + 3EC + 1 PC. This self‐constructed weak solvation effect gives Na^+^ a lower charge transfer activation energy barrier and a higher CEI density (Figure 10g). The synergistic effect of the interfacial chemistry modulation and the optimization of solvation structures also enables a high capacity of 109.7 mAh g^−1^ to be obtained, even at ‐25 °C, with a capacity retention of up to 90.8% compared to that at 25 °C.^[^ ^58^ ^]^ By diluting the ether‐based electrolyte to a concentration of 0.3 M NaPF_6_ in diethylene glycol dimethyl ether (DEGDME)/THF, the impedance (10 Ω) and polarization voltage (≈0.01 V) at −20 °C were reduced compared to the severe kinetics observed with the conventional 1.0 m carbonate electrolyte, and Na||Na_3_V_2_(PO_4_)_3_ (NVP) cells with a diluted electrolyte at −20 °C could provide a specific capacity of ≈80 mAh g^−1^ and maintain a much lower overpotential. In this weakly solvated electrolyte with low salt concentration, a solvent‐derived SEI layer was constructed, and the moderate amount of organic components allowed for more rapid Na^+^ transport at low temperatures.^[^ ^59^ ^]^ Over‐strong cation‐anion pairs tend to precipitate salt at low temperatures, resulting in a sharp reduction of carrier ions. Therefore, the interaction between cations and anions cannot be blindly enhanced, because only the appropriate interaction will have a better effect.

Finally, functional additives are also one of the most effective measures used to change the properties of the electrolyte to obtain the desired electrochemical properties. Generally, the contents of the additives in the electrolyte do not exceed 5% (wt% or vol%), and trace amounts are used to mobilize the performance changes of the entire battery system. In addition to the improvement in controlling the lithium‐ion insertion and disinsertion processes of nano‐LiFePO_4_ materials at low temperatures by changing the content of antifreeze additives in the electrolyte, this is conducive to the application of LFP at low temperatures.^[^ ^86^ ^]^

The incompatibility between graphite anodes and PC limits its practical applications. Sulfur‐based additives are recognized for their ability to stabilize graphite in PC‐based electrolytes. Density functional theory (DFT) analysis showed that the LUMO of sulfur made it possible for PC to preferentially form a Li_2_SO_3_ and ROSO_2_Li protective layer on the graphite anode. The impedance of the SEI is usually larger at low temperatures because the combination of additives and lithium ions is stronger. Since the combination of additives and Li^+^ is stronger at low temperatures, the corresponding de‐solvation of the Li^+^ becomes more difficult, which would lead to accumulation of solvated ions on the interface. The accumulated solvated Li^+^ would be decomposed and generate inferior SEI layer that contains more organic species with higher interface impedance. When dimethyl sulfite (DMS) was used as an electrolyte additive in 1 m LiPF_6_‐EC/EMC (EC/EMC = 1/2, by weight) based quasi‐electrolyte, the batteries delivered desirable low‐temperature performance with high capacity retention of 74.28% at 0.5 C and ‐20 °C.^[^ ^60^ ^]^ The results demonstrated that DMS is weaker than 1,3,2‐dioxathiolane 2,2‐dioxide (DTD) commercial additive for combination with lithium ions, which would reduce the interphase impedance and suppress the undesirable reductive decomposition of carbonate solvents and lithium salts, so as to achieve enhanced low‐temperature performance.

**Figure 11:**
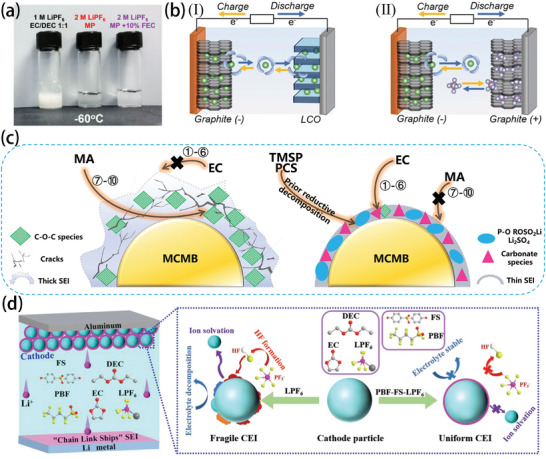
a) Physical states of different electrolytes at −60 °C. b) Schematic illustrations of I) Graphite||Lithium cobalt oxide (LCO) and II) Graphite||Graphite batteries. Reproduced with permission.^[^ ^88b^ ^]^ Copyright 2019, Wiley‐VCH GmbH. c) Illustration of the possible working mechanism of TMSP and PCS functional additives. Reproduced with permission.^[^ ^62^ ^]^ Copyright 2019, Elsevier. d) Illustration of the working mechanism with the modified electrolyte for LFP cathode. Reproduced with permission.^[^ ^63^ ^]^ Copyright 2022, Wiley‐VCH GmbH.

Fluorine‐containing additives are also widely used in low‐temperature batteries. When FEC was added to an ether‐based electrolyte (80:20 DOL/DME) with a volume ratio of 10%, the deposition and dissolution of lithium ions could be stabilized. The SEI with the FEC‐modified ether electrolyte was rich in inorganic substances (LiF, Li_2_CO_3_), and the contact between Li_2_CO_3_ and LiF can promote the accumulation of space charge on their interface. As a result, this approach delivered a higher ion carrier concentration and significantly improved lithium‐ion transportation.^[^ ^61^, ^87^ ^]^ Similarly, adding FEC to MP‐based electrolyte in a proportion of 10% can alleviate the problem of poor reversibility caused by the use of MP alone. As shown in **Figure** **11**a, the physical states of MP‐based electrolytes containing only MP and 10% FEC are different at −60 °C. The electrolyte with 10% FEC added exhibits an ionic conductivity of 1.50 mS cm^−1^ at −60 °C, and the double graphite battery maintains 84.4% of its capacity at room temperature. In addition, the presence of graphite cathodes breaks the rocking chair transmission of lithium‐ion batteries, and the increase in the ion diffusion barrier and charge transfer resistance of graphite cathodes is much smaller. The reaction process is shown in Figure 11b, and the de‐solvation step of the anionic storage cathode is omitted. Dual‐ion batteries (DIBs) require a relatively large electrolyte volume, however, and therefore cannot be compared with LIBs in terms of specific energy at the battery level.^[^ ^88^ ^]^

The addition of trimethylsilyl phosphite (TMSP) and 1,3‐propanediol cyclosulfate (PCS) binary functional additives enabled mesocarbon microbeads (MCMB)||LiNi_0.5_Mn_1.5_O_4_ to have a conductivity of 0.0817 mS cm^−1^ at ‐60 °C in the full cell. These additives can suppress the high reactivity between the methyl acetate (MA) co‐solvent and the MCMB anode, while the first reductive decomposition of the binary functional additive will alleviate the presence of the MA co‐solvent and carbonate on the surface of the MCMB anode, as illustrated in Figure 11c. It also can inhibit the surface destabilization of MCMB and reduce dendrite formation.^[^ ^62^ ^]^

When CO_2_ was first used as an electrolyte additive,^[^ ^89^ ^]^ the application of inorganic additives came into being. They improve the electrolyte performance and can be comparable to organic additives, but the inorganic additives have the advantages of high safety, low price, easy‐to‐obtain raw materials, etc., which not only improves the performance of the SEI but also regulates the CEI. Since SEI/CEI layers formed by organic additives usually suffer from low ionic conductivity and high interfacial impedance, attention has turned to inorganic salt‐based additives.^[^ ^90^ ^]^ such as lithium difluoro(bisoxalato)phosphate (LiDFBOP),^[^ ^91^ ^]^ LiFMDFB, and AgNO_3_.^[^ ^48^ ^]^

In addition to such an additive promoting the compatibility of the electrolyte and electrode, and inducing the stable formation of SEI, the additive could also alter the solvation structure of the electrolyte to improve the low temperature performance of the electrodes.^[^ ^92^ ^]^ Research has shown that the weakening of the Li^+^‐solvent interaction is the root cause of the graphite electrode's excellent compatibility with electrolyte.^[^ ^93^ ^]^ The additives have been demonstrated to function cooperatively, altering the Li^+^ coordination structure and Li^+^ solvent‐pair interactions within the electrolyte. Cheng also pointed out that additives with weak coordination capability (e.g., EC, VC) are not sufficient to make the PC‐ and ether‐based electrolytes compatible with graphite, although they can also change the Li^+^ solvation structure.^[^ ^23^ ^]^ This is because additives with weak coordination ability are unable to effectively weaken the Li^+^‐solvent interaction. Subsequently, additives to change the solvation sheath appeared one after another. The addition of 4,4ʹ‐sulfonyldiphenol (FS) and perfluoro *n*‐butylsulfonyl fluoride (PBF) changed the solvent sheathing structure of the carbonate electrolyte, thus yielding an LiF‐rich SEI interface layer and stabilizing the LFP cathode (Figure 11d). Lithium‐ion batteries using the PBF‐FS‐LPF_6_ electrolyte maintained 90% of their capacity after 100 cycles, even at −40 °C.^[^ ^63^ ^]^ In 2024, Wang et al. further determined that the dynamic evolutionary interface of the electrolyte configuration at the Li/electrolyte interface essentially determines the structural composition of the SEI.^[^ ^12^ ^]^ Therefore, we need further exploration of solvated configurations formed by intermolecular forces that cannot only form favorable SEI layers, but also improve the compatibility of electrolytes and electrodes.

These studies indicate that the exploration of additives should not only be concerned with their ability to form stable SEI layers, but also consider the intermolecular forces and solvated structure or interfacial model (from the point of view of desolvation). It has been revealed that conductivity is not the most fatal influence on battery performance, and the balance of ion–ion and solvent–ion interaction forces is fundamental to an in‐depth study of solute salts of non‐aqueous low‐temperature electrolytes.^[^ ^94^ ^]^

### 3.2 Aqueous Electrolytes

**Figure 12:**
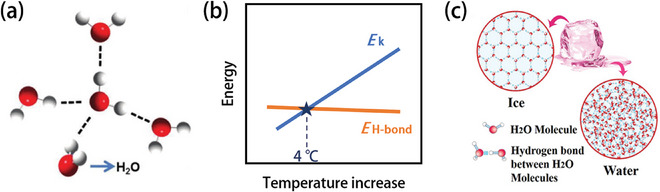
a) Illustration of tetrahedral water molecules connected by HBs. Reproduced with permission.^[^ ^102^ ^]^ Copyright 2023, Wiley‐VCH GmbH. b) Temperature‐dependent energy in pure water, *E*_k_, kinetic energy, and *E*_H‐bonds_, the energy of the HBs. c) Illustration of the HBs in water and ice. Reproduced with permission.^[^ ^8b^ ^]^ Copyright 2020, Published by Wiley‐VCH GmbH.

Non‐aqueous electrolytes with organic solvents typically exhibit high material prices and are flammable. As a result, aqueous electrolytes have been developed as feasible alternatives.^[^ ^95^ ^]^ Water as a solvent in aqueous electrolytes is the main reason for the high freezing point of this type of electrolyte. Water contains a large number of HBs.^[^ ^96^ ^]^ HBs are coordination bonds between lone pairs of electrons in N, O, and F on the one hand and positively charged hydrogen atoms on the other, rather than shared electron pairs.^[^ ^97^ ^]^ From the perspective of the chemical composition, each chemical formula contains a polar water molecule with two hydrogen atoms and one oxygen atom, and the intrinsic electrical structure of the H_2_O molecule confers on H and O a lack of electrons and a sufficient number of electrons, respectively, to make them both acceptors and donors for HBs. The O atoms possess a negatively charged side, while the H atoms have a positively charged side, which provides the conditions for the formation of HBs. HBs in water are formed between O atoms and H atoms of adjacent H_2_O molecules through electrostatic interactions.^[^ ^98^ ^]^ Subsequently, as the viscosity of liquid water increases with a decreasing temperature, the HB restricts the vibration of water molecules, and several persistent HBs accidentally appear at the same location where they form polyhedral structures, and successive random HB networks give rise to supramolecular structures or water clusters in nature that evolve into stable initial ice nuclei.^[^ ^99^ ^]^ The initial formation stage of ice nuclei relies on water molecules with tetrahedral coordination structures to grow into stacked hexagonal sequences to promote the formation of long‐range ordered crystalline structures at low temperatures. Below 0 °C, during ice formation, an additional 0.52 HBs are formed per water molecule.^[^ ^8^, ^17^, ^98^, ^99^ ^]^ Adjusting HBs can inhibit the freezing of water from the initial ice nucleation stage. From a theoretical perspective, a single water molecule can engage in effective interactions with up to four neighboring water molecules through hydrogen bonding, forming self‐associated water clusters, as shown in **Figure** **12**a. Although the average kinetic energy of water molecules is closely related to the ambient temperature, the average bond energy of O─H···O is a constant value. When the ambient temperature is higher than 4 °C, its kinetic energy is greater than the HB interaction. The migration of water molecules is not restricted by the HB network, and liquid water has graceful mobility. When the temperature decreases, its kinetic energy decreases linearly with the decrease in temperature, and the probability of HB formation gradually becomes higher than its probability of rupture. When the temperature is lower than 4 °C, the movement of water molecules is gradually restricted due to the enhancement of total HB interactions (Figure 12b). In this system, the viscosity increases and the fluidity decreases.^[^ ^8^, ^99^, ^100^ ^]^ Eventually, liquid water completely solidifies into ice when the temperature is lower than the freezing point, and the original local short‐range ordered structure gradually transforms into a long‐range ordered network. Finally, solid water ice is formed (Figure 12c).^[^ ^101^ ^]^

In summary, it can be concluded that the presence of HB between water molecules is the main reason for its higher freezing point, which provides a direction for the development of unfrozen aqueous electrolytes below zero degrees. Thus, the freezing point of aqueous electrolytes is lowered by preventing the formation of a regular hydrogen bonding network during cooling. This primarily enhances low‐temperature energy storage by increasing the ion‐water molecule interaction force, thereby weakening the water molecule interaction force and reducing the freezing point.^[^ ^13^, ^103^ ^]^ Although the weakening of HB strength is conducive to the improvement of low temperature performance, the increase of HB strength is conducive to the extension of the voltage window of the electrolyte. The strength of HB interaction can be regarded as a metric, which also indicates that we should seek a balance in the aqueous electrolyte.^[^ ^104^ ^]^ The electrolyte composition, cathode, anode, and electrochemical performance of representative state‐of‐the‐art reports on aqueous low‐temperature rechargeable batteries have been summarized in **Table** **2**.

**Table 2:** Summary of state‐of‐the‐art reports on aqueous low‐temperature rechargeable batteries.

Electrolyte	Electrodes	Operating temperature [°C]	Capacity [mAh g^−1^]	Rate [C]	Cycles	Reference
2 m NaClO_4_, 0.3 m DMSO	AC||NTP	−50	60	0.5	100	[105]
1 m NaNO_3_, glycerol/DI (2:1)	Ni_2_ZnHCF||PTCDI	−10	40	100 mAg^−1^	N/A	[106]
NaClO4_1.7_‐H_2_O_5.5_‐FA_5.81_	AC||Polymer	−50	80	4	8000	[107]
Zn(OTf)_2_, TMP/H_2_O	Zn||VO_2_(B)	0	200	1 A⋅g^−1^	1200	[108]
5 m H_2_SO_4_	Pb||PCHL‐rGO	−70	50	0.5 A g^−1^	500	[109]
3.86 m CaCl_2_ + 1 m NaClO_4_	Na_2_CoFe(CN)_6_||AC	−30	64.6	1	1000	[102]
7.5 m ZnCl_2_	Zn||Polyaniline	−70	84.9	0.2A g^−1^	2000	[98a]
3.5 m Mg(ClO_4_)_2_ + 0.5 m NaClO_4_	AC||NaTi_2_(PO_4_)_3_@C	−60	23.2	8	10 000	[110]
2 m Zn(CF_3_SO_3_)_2_	Zn||V_2_O_5_	−30	285.0	0.5A g^−1^	1000	[111]
17 m NaClO_4_	NVP||NVP	−40	16	0.3 mA cm^−2^	N/A	[112]
4 m Zn(BF_4_)_2_	Zn|| Tetrachlorobenzoquinone	−60	87.6	0.1	50	[113]
2 m HBF_4_ + 2 m Mn(BF_4_)_2_	Carbon felt||Alloxazine	−60	120	4	500	[114]
7.6 m ZnCl_2_ + 0.05 m SnCl_2_	Zn||VOPO_4_	−50	>100	1/3	200	[115]
45 m ZnBr_0.5_Cl_1.5_ + 1 m Zn(OAc)_2_	Zn/GFF||graphene	N/A	500	1 A g^−1^	500	[116]
Zn(ClO_4_)_2_·6H_2_O:SN (1:8)	Zn||PDB	−20	∼50	0.15	N/A	[117]
Zn(ClO_4_)_2_·6H_2_O:SL (1:6)	Zn||PANI	−30	73	0.3 A g^−1^	500	[118]
2 m ZnSO_4_·7H_2_O‐ DMSO/H_2_O (60%)	Zn||MnO_2_	−20	≈70	1	300	[119]
1 m Zn(CF_3_SO_3_)_2_, ACN/H_2_O (72:28)	Zn||V_2_O_5_	−40	69	1 A g^−1^	1000	[120]
Zn_4_SO_4_(OH)_6_·xH_2_O:MeOH (1:1)	Zn||PANI	−10	100	5A g^−1^	2000	[121]

AC, activated carbon; GFF, graphene fiber fabric; PDB, poly(2,3‐dithiino‐1,4‐benzoquinone); PANI, polyaniline.

#### 3.2.1 Weakening Solvent–Solvent Interactions

Adjustment of the solvent is an effective way to reduce the freezing point of the electrolyte. We can think of the O─H bonds in water molecules as hydroxyl groups, which break the HB network of water molecules by providing donors or acceptors of hydroxyl groups in the electrolyte or providing substances that have strong HB interactions with water molecules,^[^ ^122^ ^]^ thereby reducing the viscosity and freezing point of the electrolyte at low temperatures or achieving the goal of low‐temperature operation through new methods such as eutectic, ion solvation, and water‐in‐salt (WIS). In addition to making adjustments to the HB network in the electrolyte, the involvement of Lewis acids and bases, as well as van der Waals forces, will also contribute to varying degrees to the regulation of electrolyte components.^[^ ^123^ ^]^ The effect of freezing point depression can be obtained by adjusting the physical and chemical properties by force.^[^ ^124^ ^]^ After adjusting the solvent, the electrolyte should maintain the advantages of the aqueous electrolyte such as high conductivity as much as possible, and at the same time, eliminate the impact of its disadvantages on the performance of the electrolyte as much as possible.

**Figure 13:**
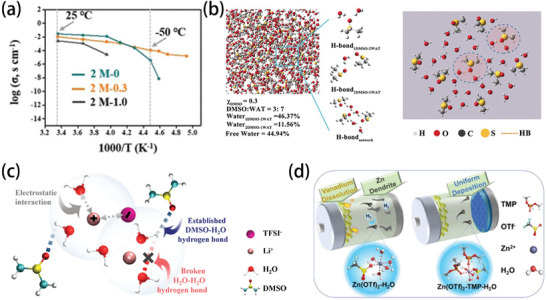
a) Temperature dependence of the ionic conductivity.^[^ ^134^ ^]^ b) Conformation analysis of the system with DMSO mole fraction (χ_DMSO_) = 0.3 and local structure of the χ_DMSO_ = 0.3 system from MD simulations. Reproduced with permission.^[^ ^134^ ^]^ Copyright 2019, Wiley‐VCH GmbH. c) Schematic illustration of possible supramolecular interactions between LITFSI, DMSO, and water. Reproduced with permission.^[^ ^126^ ^]^ Copyright 2021, Elsevier. d) Schematic illustration of the solvation structure in the aqueous battery. Reproduced with permission Reproduced with permission.^[^ ^108^ ^]^ Copyright 2022, Elsevier.

As a stable sulfur‐containing compound, dimethyl sulfoxide (DMSO) is miscible with water in any proportion as an HB acceptor, and the freezing temperature can reach −140 °C.^[^ ^125^ ^]^ As mentioned above, when DMSO is added as an additive to 2 m NaClO_4_, the freezing point can be significantly lowered. In the new 2 M NaClO_4_ + 0.3 M DMSO electrolyte, the freezing point can reach below −130 °C, while the conductivity of 0.11 mS cm^−1^ can be reached at ‐50 °C. Spectral investigations and molecular dynamics (MD) simulations revealed that stable HBs can form between DMSO and water molecules, with DMSO acting as the HB acceptor and water as the donor. It can be seen from **Figure** **13**a that at different concentrations, the ionic conductivity of 0.3 M DMSO has a lower dependence on temperature. As shown in the molecular dynamics simulation in Figure 13b, more water molecules will be trapped in DMSO‐water agglomerates than in water‐water agglomerates, which prevents water molecules from forming an ordered HB network, and since the network of HBs is the geometric basis of crystallization, thereby achieving the effect of lowering the freezing point.^[^ ^105^ ^]^ In the new electrolyte proposed by Tang et al., which shows supramolecular induction of LiTFSI in DMSO and water cosolvent systems in a novel electrolyte, the O atoms in DMSO can also form strong HBs with H atoms in H_2_O (Figure 13c), so it also partially suppresses the hydrogen evolution reaction. The operating temperature range of the new electrolytic supercapacitor with this electrolyte can range from −40 to 90 °C.^[^ ^126^ ^]^

Methanol (Me), ethanol (Et), ethylene glycol (Eg), and glycerin (Gl) are frequently employed as industrial antifreeze agents due to their miscibility with water, forming homogeneous solutions. Therefore, we mixed alcohols as additives into the electrolyte to break through the freezing point limit.^[^ ^127^ ^]^ Gl with three hydroxyl groups has a strong ability to destroy the HB network and adjust the electrolyte structure. In addition, HBs between glycerol and water are formed in this regard, inhibiting the activity of water protons, and the freezing point drops to below −80 °C in the case of 2:1 glycerol and deionized water. Achieving a nickel‐zinc hexacyanoferrate||3,4,9,10‐perylenetetracarboxylic diimide (Ni_2_ZnHCF||PTCDI) full battery capacity of 40 mAh g^−1^ at −10 °C, which is equivalent to 80% of the capacity retention rate at 25 °C.^[^ ^106^ ^]^ In the latest polyethylene glycol study, Xu et al., by precisely adjusting the molecular weight and addition ratio of polyethylene glycol, anchored the active water and ions, so that Zn^2+^, solvent water, and ether bond groups locally aggregated to form CIPs. Under the extreme temperature of −30 °C, the full battery, where LA‐Zn(OTf)_2_ is Lewis acid zinc trifluoromethanesulfonate and NVO is NaV_6_O_15_, achieved 2000 stable cycles at 0.5 A g^−1^. The capacity retention rates were 80.5%.^[^ ^128^ ^]^ Similarly, PEG as an auxiliary agent breaks the intrinsic strong HB of water through global weak PEG–H_2_O interaction, strengthening the O‐H covalent bond of water and the coordination with Zn^2+^. This synergistic effect greatly reduces water activity, in an optimized electrolyte, the ZIBs not only deliver excellent cycling performance, with a low attenuation rate of 0.0009% per cycle over 20 000 cycles, but also show stable performance over a wide temperature range of 40 to −40 °C. This study provides valuable insights into the rational design of ZIBs electrolytes.^[^ ^129^ ^]^ Although the DMSO and alcohols discussed above have definite coordination with water molecules through S═O and O─H, respectively, reconstructing the HB network in the system. Other functional groups are wasted to a certain extent, and even the ion conductivity is also affected by steric hindrance. Therefore, formamide (FA), a strongly polar organic solution with high polarity and high utilization of functional groups, is another good choice.

The liquid–solid temperature range (LSTR) of a mixture of FA and water, which is both an HB acceptor and a donor, is much lower than the melting point of FA (2 °C) or H_2_O. The low LSTR of the FA‐H_2_O mixture meets the condition for the preparation of low temperature electrolyte.^[^ ^130^ ^]^ Theoretical calculations and experimental results indicate that FA possesses sufficient active sites to coordinate with water molecules, thereby disrupting their direct interactions and impeding the formation of long‐range ordered structures at below‐zero temperatures. An ultra‐low freezing point (<−50 °C) was also achieved in an ultra‐low‐temperature aqueous sodium ion hybrid battery (ASIHB) electrolyte of (NaClO_4_)1.7‐(H_2_O)5.5‐(FA)5.81 using the strong polar solvent FA as a co‐solvent for (NaClO_4_)1.7‐(H_2_O)5.5.^[^ ^107^ ^]^ The newly formed hybrid system exhibits strong interaction between water and FA molecules due to the strong electron absorption of carbonyl and hydroxyl groups, such as C═O···HO and NH···OH, reconstructing the HB network and effectively limiting the vibration state of water molecules, thus reaching an ultra‐low freezing point.

Trimethyl phosphate (TMP) has also been reported to modify the solvation structure of electrolytes. The bond energy between TMP and Na^+^ is much lower than the bond energy between water molecules and Na^+^. Therefore, in terms of cationic effects alone, water molecules are strongly constrained around Na^+^, thus inhibiting the formation of an HB network in water, although TMP can induce significant electronic modulation of the electrolyte bonding environment. This allows Na^+^‐H_2_O to bind more strongly to the freely migrating TMP. An electrochemical double‐layer capacitor with a mixed electrolyte of 3 M NaClO_4_ in water and TMP not only operated over a wide voltage range of 0–2.4 V at temperatures from −20  to 60 °C, but also has excellent rate capability and cycling stability. For example, at 25 °C, the capacitance retention rate was 83% after 100 000 cycles.^[^ ^131^ ^]^ In zinc‐ion batteries, the introduction of TMP or TEP phosphate into the water‐electrolyte system also helps to reshape the Zn^2+^‐solvation shell, thus inhibiting H_2_O activity and water‐induced side reactions to avoid dendrite growth.^[^ ^108^, ^132^ ^]^ The number of Gutmann donors in TMP is 23 kcal mol^−1^, which is much higher than for H_2_O. Thus, when TMP acts as a proton acceptor and mixes with water molecules, it can break up the HB network and add the first solvation sheath of Zn^2+^, replacing the involvement of water to form a solvated structure, as in Figure 13d. A Zn||VO_2_(B) full cell in the environment of Zn(OTf)_2_ electrolyte into which TMP had been introduced exhibited a cycling stability of 1200 at 0 °C, 1 A g^−1^.^[^ ^108^ ^]^ The donor number (DN), introduced by Gutmann, is a critical parameter for electrolytes, which strongly influences the solvation energy and structure of the cations. Therefore, solvents with higher DN values are more likely to form solvated structures with Zn^2+^.^[^ ^133^ ^]^ This is the fundamental reason why this water‐soluble additive can lower the freezing point.

#### 3.2.2 Strengthening Ion–Solvent Interactions

Two primary interactions influence the HBs between water molecules: the metal ion‐H_2_O and anion‐H_2_O interactions.^[^ ^135^ ^]^ Since the initial demonstration that ions can alter the structure and dynamics of HBs in water molecules, ion‐driven modification of the tetrahedral structure of water molecules has been a topic of research and will be for decades to come.^[^ ^136^ ^]^ The cooperative interaction between ions and water molecules can disrupt the intrinsic HB network, leading to changes in the macroscopic properties of water molecules (e.g., freezing point, surface tension, and viscosity).^[^ ^136^, ^137^ ^]^ The polarization abilities of cations in different electrolytes to water molecules are different, however, which is also a key factor affecting the freezing point of electrolytes. In the ionic potential formula *Φ = Z/r*, where *Z* is the number of ionic charges and *r* is the ionic radius, we can derive from the ionic potential formula that the higher the charge number and the smaller the size of the cation, the higher the ion potential and the stronger its corresponding polarization ability, the stronger the electrostatic force of attraction with the O atom inside the water molecule will be, which will have a strong impact on the distribution of the surrounding water molecules and change its original HB network.^[^ ^138^ ^]^ For the primary hydration process of different cations, the numbers of polarized water molecules forming the lowest energy level of the process are different, which may be related to the amount of charge and the electron distribution of the cations. Therefore, when the anions are the same, cations with higher charges can perform better at lower temperatures.^[^ ^139^ ^]^ Rechargeable batteries are categorized into types such as proton, lithium‐ion, zinc‐ion, sodium‐ion, etc., which are based on the primary carriers present in their electrolytes. For different battery systems, the mechanisms and performances of antifreeze electrolytes are slightly different.

**Figure 14:**
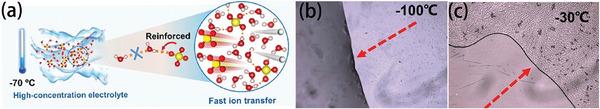
a) The mechanism by which H_2_SO_4_ electrolyte prevents freezing. Reproduced with permission.^[^ ^109^ ^]^ Copyright 2021, Wiley‐VCH GmbH. b) Polarizing microscope observations of 3.86 m CaCl_2_ + 1 m NaClO_4_ electrolyte at −100 °C and c) 1 m NaClO_4_ electrolyte at −30 °C, respectively. Reproduced with permission.^[^ ^102^ ^]^ Copyright 2022, Wiley‐VCH GmbH.

Cations could break up HB formation by binding water molecules together. The protons in the cell can diffuse very quickly, which offsets some of the effects of slow redox reactions at low temperatures.^[^ ^140^ ^]^ Yue et al. applied the proton transport mechanism. The good structure of the p‐chloranil/reduced graphene oxide (PCHL‐rGO) battery that they used was conducive to the H^+^ coordination/non‐coordination reaction and achieved faster kinetics. Meanwhile, the anions in H_2_SO_4_, which provided H^+^, could regulate interactions with water molecules through SO_4_^2−^ and lower the freezing point of the electrolyte (**Figure** **14**a). The Pb||PCHL‐rGO battery could still operate stably at −70 °C.^[^ ^109^ ^]^

Sodium salt has also been widely studied because of its large Na^+^ radius, which can be dissolved in low‐dielectric‐constant solvents.^[^ ^141^ ^]^ Because of the strong interaction between CaCl_2_ and water molecules, which can adjust the HB ratio in the water molecules, thus destroying the original HB network and lowering the freezing point of the optimized electrolyte, Zhu et al. added it as a solute additive to 1 m NaClO_4_ by 3.86 m CaCl_2_ (m: mol kg^−1^). In this electrolyte, Na^+^ acts as a carrier ion and Ca^2+^ is electrochemically inert during the cycling.^[^ ^102^ ^]^ No obvious ice crystals were observed in the optimized electrolyte by optical microscopy during cooling from −40 to −100 °C, whereas 1 M NaClO_4_ without added CaCl_2_ already showed significant solidification at ‐30 °C (Figure 14b,c). In this electrolyte, the Na_2_CoFe(CN)_6_ activated‐carbon all‐inorganic battery had a high discharge capacity of 74.5 mAh g^−1^ at 1 C, superior rate capability, and ultra‐stable performance up to 6000 cycles at 10 C and at −30 °C. This also means that adding the right concentration of inorganic solvent to the water has a very good effect toward lowering the temperature.

Zhang et al. adjusted the electrolyte structure to destroy the original HB network in the ZnCl_2_ solution, thereby inhibiting the freezing of water and reducing the solid‐liquid transition temperature of the aqueous electrolyte from 0 °C to −114 °C. In an electrolyte of 1‒7.5 m (mol kg^−1^), Tt (solid–liquid transition temperature) gradually decreased as the concentration *C*_ZnCl2_ increased, dominated by HB breaking, and as the concentration exceeded 7.5 m, the freezing point begins to rise, dominated by increased ionic interactions. This suggests that there is usually an equilibrium between strong ion–ion and water–ion interactions. There is a key *C*_ZnCl2_, that balances the interaction of HBs and ions, as shown in Figure 14d, thereby achieving the lowest freezing point for low‐temperature electrolytes. The 7.5 m ZnCl_2_‐based low‐temperature electrolyte allowed their Zn||polyaniline battery to deliver excellent low temperature performance ranging from −90 to 60 °C, covering the most extreme temperatures on the Earth's surface. This battery exhibited robust performance at −70 °C (84.9 mAh g^−1^) and maintained stability over 2000 cycles with nearly 100% capacity retention.^[^ ^98a^ ^]^ There are times when the size of the solvated shell layer caused by the concentration polarization of Zn^2+^ becomes large and unfavorable for ion transport, which requires the addition of co‐solvents such as ACN to promote the dissociation of ion pairs and attenuate the ion–ion interactions, to achieve the effect of high ionic conductivity even at low temperatures.^[^ ^120^ ^]^ Zhu's team developed a new mixed 3.5 m Mg(ClO_4_)^2+^ 0.5 m NaClO_4_ electrolyte, which had an ultra‐low freezing point close to −120 °C, and the conductivity could reach 4.6 mS cm^−1^ at −80 °C. They used the high ionic potential and strong polarization effect of Mg^2+^ to lower the freezing point of the electrolyte.^[^ ^110^ ^]^

In order to increase the electrochemical window of water‐based electrolytes, electrolytes mixed with high‐concentration double salt mixtures have been extensively studied after it was demonstrated that water molecules have strong interactions with cations and that water activity is further reduced. Gao et al. designed a dual‐salt electrolyte containing a high concentration of a supporting salt, 1 m Al(OTF)_3_ + 15 m LiOTF, where OTF stands for trifluoromethane sulfonate, which greatly expanded the electrochemical window to 4.35 V and reduced polarization. The spectroscopic results and kinetic simulations of this experiment also reveal that the superior performance originates from the evolution of the solvation sheath and significantly reduced water activity of Al^3+^ in high‐concentration aqueous electrolytes (Figure 14e).^[^ ^142^ ^]^ Adding different types of solutes to the aqueous electrolyte turns the solutes in the electrolyte into binary and other multi‐component systems, which can also lower the freezing point of the electrolyte, greatly improving its performance.

Apart from the impact of cations on the electrolytes, as discussed above, anions also play a vital role in the performance of the electrolytes. Anions with different binding energies to water molecules or anions with different HB receptor elements (e.g., N, F, O) can bind to water molecules and alter their activity.^[^ ^143^ ^]^ The radius of ions is an important factor in determining the strength of hydration. Although the radii of anions are larger than those of cations, anions can interact with hydrogen atoms in water molecules, because water is both a HB donor and an HB acceptor, so the HB network can be disrupted by anions becoming HB acceptors or forming hydration structures under the action of static electricity.^[^ ^105^ ^]^ When the cation and anion have the same radius, the hydration of the anion is stronger, but in practice, the anion has weaker hydration due to its larger radius.^[^ ^1^, ^144^ ^]^ Moreover, the types and concentrations of anions in the electrolyte directly influence the coordination of ions and the Gibbs free energy. Anion modification enhances the ion desolvation effect, thereby improving the kinetics of solid–liquid interface reactions and facilitating rapid ion transfer. This leads to lower dissolution energy and a stable solid–liquid interface.^[^ ^145^ ^]^ It has also been reported that different anion types have different effects on HBs in water, and the order of effects is the Hofmeister series.^[^ ^111^, ^146^ ^]^

**Figure 15:**
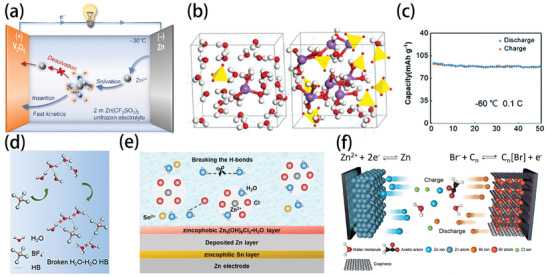
a) Diagrammatic representation of a Zn||V_2_O_5_ battery with hydrated Zn^2+^ insertion. Reproduced with permission.^[^ ^111^ ^]^ Copyright 2021, American Chemical Society. b) MD simulations of the electrolyte configurations of 2 m NaClO_4_ and 17 m NaClO_4_. Atom colors are designated as follows: Na (purple), O (red), H (white), and Cl (yellow). Reproduced with permission.^[^ ^112^ ^]^ Copyright 2020, Elsevier. c) The extended cycling stability of the Zn||TCBQ battery at −60 °C. Reproduced with permission.^[^ ^113^ ^]^ Copyright 2021, Royal Society of Chemistry. d) Schematic illustration depicting a plausible mechanism for disrupting hydrogen bond networks in water molecules by introducing BF_4_^−^ anions. Reproduced with permission.^[^ ^114^ ^]^ Copyright 2021, Wiley‐VCH GmbH. e) High‐capacity aqueous dual‐ion battery enabled by WSOE_45_‐1. Reproduced with permission.^[^ ^115^ ^]^ Copyright 2021, Wiley‐VCH GmbH. f) Schematic illustration of the structure of the electrolyte and the electrolyte‐electrode interphase. Reproduced with permission.^[^ ^116^ ^]^ Copyright 2021, Wiley‐VCH GmbH.

Even Zn^2+^, which has the strongest solvation force with water, has a solvation capacity that is affected by the type of anion. Anions with higher electrostatic potentials (ESPs) can lead to larger zinc‐water coordination numbers. In a basic study of five commonly used zinc salts with different anions, ZnSO_4_, Zn(NO_3_)_2_, ZnCl_2_, ZnI_2_, and Zn(CF_3_SO_3_)_2_, the negative electrostatic potentials of the five anions were ranked as SO_4_^2−^ < NO^3 −^ < Cl^−^ < I^−^ < CF_3_SO_3_^−^. SO_4_^2−^ has the lowest ESP value and has a strong interaction with Zn^2+^, resulting in Zn‐O_SO4_^2−^. This leads to easy participation of anions in the coordination shells of cations, which would crowd out the water in the solvated shells and attenuate the cation‐induced HB breaking. In contrast, the electrolyte consisting of CF_3_SO_3_^−^ with weak interaction with H_2_O/Zn^2+^ could reach a low freezing point of −34.1 °C and had a high ionic conductivity of 4.47 mS cm^−1^ at −30 °C (**Figure** **15**a).^[^ ^111^ ^]^ Since the relative destabilizing ability of inorganic ions with respect to bulk water molecules is ranked as SO_4_^2−^ < TFSI^−^ < ClO_4_^−^, ClO_4_^−^, the salt exhibits a strong tendency to disrupt water molecule networks and can alter solvation structures through ion aggregation. As shown in Figure 15b, NaClO_4_ forms a complex ion network with water molecules, and a solution with a high concentration can better destroy the original HB network. One team took advantage of this feature and used water‐in‐salt (WIS) electrolysis of 17 m NaClO_4_ to solve the low‐temperature freezing problem. Their prepared NVP| NaClO_4_ |NVP had a high capacity of 16 mAh cm^−3^ and a high retention rate of 88% when cycled at −40 °C for 30 times.^[^ ^112^ ^]^

Introducing anions with highly electronegative elements (e.g., N, O, F) as HB receptors to break the existing HB network in water and lower the freezing point is considered to be an effective strategy.^[^ ^147^ ^]^ Sun and colleagues found through spectral measurements and theoretical calculations that the introduction of BF_4_^−^ anions could destroy the HB network in the original water molecules by forming OH‐F HBs, thereby producing ultra‐low freezing points. Their 4 m Zn(BF_4_)_2_ based electrolyte allowed their Zn//tetrachlorobenzoquinone (TCBQ) battery to demonstrate outstanding electrochemical performance across a wide temperature range from −95  to 25 °C, achieving a record capacity of 63.5 mAh g^−1^ and energy density of 76.2 Wh kg^−1^ at −95 °C. It could also maintain long‐term stable cycling at ‐60 °C (Figure 15c). Their electrolyte solution of 2 m HBF_4_ + 2 m Mn(BF_4_)_2_, which also contained BF_4_^−^, also had good low‐temperature performance, with a freezing point below −160 °C, proving the formation of OH‐F HBs (Figure 15d). It had a high ionic conductivity of 0.21 mS cm^−1^ at −70 °C.^[^ ^113^, ^114^ ^]^

Halogen anions can also interact strongly with water molecules, thus breaking the water‐water connection. In a 7.6 m ZnCl_2_ solution, in which the main component was [Zn(H_2_O)_2_Cl_4_]2^−^, Cl^−^ was not only involved in the solvation sheath of Zn^2+^ but also formed strong H_2_O–Cl^−^ interactions with water molecules, as shown in Figure 15e. The temperature range of the electrolyte reached 20 to −70 °C when 0.05 m of SnCl_2_ was added.^[^ ^115^ ^]^ In an overconcentrated solution of 45 m ZnBr_0.5_Cl_1.5_ + 1 M Zn(OAc)_2_ water salt oligomer electrolyte (WSOE). In the following, WSOE_X_‐Y is used, where X is the total mass molar concentration of ZnCl_2_ and ZnBr_2_, and Y is the mass molar concentration of Zn(OAc)_2_. Throughout the entire range of detection temperatures (−80 to 40 °C), no corresponding salt crystallization or water freezing peaks were observed for WSOE_45_‐1. In addition, the glass transition temperature (*T*_g_) of WSOE_45_‐1 was measured at ≈−70 to −60 °C. This phenomenon arises from the formation of the Br^−^/Cl^−^···H pair in WSOE_45_‐1 as an alternative interaction to the O···H pair, which leads to a reduction in the frequency of unpaired O─H stretching. Such interactions reconstruct the solvent framework and link halogen anions to water molecules, forming fragments of aqueous salt oligomers. The reaction mechanism of the high‐capacity water dual‐ion battery realized by WSOE_45_‐1 is shown in Figure 15f.^[^ ^116^ ^]^

The low‐temperature contribution of anions to the electrolyte is mainly to break HBs to become HB acceptors or form solvation structures with metal ions to relieve low‐temperature freezing. In general, the design idea for electrolyte solutes at low temperatures is mainly to achieve the goal of a low freezing point by destroying the HB network through the different effects of anions and cations on water molecules.

The eutectic electrolyte is a typical strong bonding ionic‐solvent electrolyte.^[^ ^13b^ ^]^ Eutectic electrolytes are essentially a class of deep eutectic solvents (DESs). They are composed of two or more compounds that offer stronger and richer intermolecular reactions (e.g., HB interactions, Lewis acid‐base interactions, and van der Waals interactions) rather than individual components, and this unique formation mechanism gives eutectic hybrid electrolytes a distinctive chemical environment and complex structure that allows them to operate over a wide range of temperatures.^[^ ^148^ ^]^ Due to the presence of anionic and cationic complexes, eutectic electrolytes also have the properties of incombustibility, easy synthesis, and structural flexibility that other electrolytes do not have. Adjusting the ratio of components to achieve the effect of enhancing molecular interactions thereby lowers the freezing point of the system, which is why DES is used in low‐temperature electrolytes.^[^ ^123^, ^149^ ^]^

**Figure 16:**
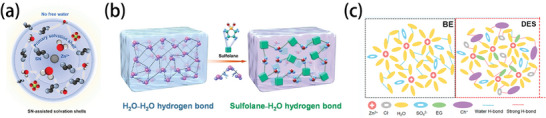
a) Recombinant solvated shell structure of Zn^2+^. Reproduced with permission.^[^ ^117^ ^]^ Copyright 2020, Elsevier. b) Schematic illustration of the HB reconstruction involving sulfolane. Reproduced with permission.^[^ ^150^ ^]^ Copyright 2022, Wiley‐VCH GmbH. c) Solvent structures in BE (baseline electrolyte, left) and DES (right). Reproduced with permission.^[^ ^151^ ^]^ Copyright 2023, American Chemical Society.

Recently, DESs have received increasing attention due to their cryogenic prospects. When the molar fraction of lithium salt reaches 0.2, the eutectic point is located at a eutectic temperature of −72 °C in the case of LiTFSI and N‐methyl acetamide mixtures.^[^ ^152^ ^]^ This provides a framework for the normal operation of DES electrolytes at low temperatures. When the molar ratio of DMSO to H_2_O is 1:2, the melting point of the thus‐formed eutectic mixture is as low as −140 °C. When a certain concentration of LiTFSI is dissolved, the supercapacitor battery containing it can operate at temperatures as low as −35 °C and as high as 65 °C. It thus operates in the extreme ambient temperature range and exhibits excellent energy and power density.^[^ ^153^ ^]^ According to a new aqueous eutectic electrolyte proposed by the above report, by coupling a hydrated zinc salt (Zn(ClO_4_)_2_·6H_2_O) with a neutral ligand (succinonitrile (SN)), SN replaces most of the water molecules in the primary solvated shell layer of Zn^2+^, as in **Figure** **16**a, leading to a solvation transition from [Zn(OH_2_)_6_]^2+^ to [Zn(OH_2_)_x_(SN)_y_]^2+^, and the eutectic nature of the (Zn(ClO_4_)_2_‐6H_2_O/SN ratio of 1:8 (denoted as ZS)) also provides a rich set of internal interactions (e.g., H_2_O‐SN and H_2_O‐ClO_4_), and those water molecules that are substituted remain hydrated in the eutectic structure, all of which make free water scarce. This zinc–organic battery, using an aqueous eutectic electrolyte, delivered exceptional cyclability with minimal capacity degradation (0.004% degradation after 3500 cycles) and excellent low‐temperature performance.^[^ ^117^ ^]^ The strong synergistic interaction between sulfolane (SL) and Zn^2+^ triggers a deep eutectic effect, resulting in a Zn(ClO_4_)_2_‐6H_2_O:SL = 1:6 eutectic with a wide operating temperature window from −40 °C to room temperature.^[^ ^118^ ^]^ Relative to water molecules, SL can provide more hydrogen‐bonding acceptors, which facilitates the breaking of the intrinsic H_2_O–H_2_O HB and the formation of a new butyl sulfone‐water network, as shown in Figure 16b. The water activity was suppressed, and the new strategy using Zn(TFSI)_2_‐sulfolane‐H_2_O deep eutectic solvent achieved an ultra‐low freezing point of −80 °C.^[^ ^150^ ^]^ The strong HBs formed between the eutectic components formed by choline chloride (ChCl) and ethylene glycol (EG) with water replace the original HBs between water and water, which leads to the structural remodeling of Zn^2+^ solvation and the remodeling of the HB network in the electrolyte (Figure 16c). The significant reduction in HBs results in a lower freezing point of the electrolyte. Strong HBs formed between the introduced eutectic components and water molecules disrupt the weaker HBs in the original water molecule network, which contributes to the ultra‐low freezing point and high ionic conductivity of 1.7 mS cm^−1^ at −40 °C.^[^ ^151^ ^]^

The introduction of organic solvents to enable metal ions to form corresponding solvated structures with them is another effective design technique to extend the temperature range, which is essentially a method of lowering the temperature by strengthening the HBs between organic solvents and solvated structures formed by organic solvents with metal ions and water molecules, thereby causing damage to the original HB network of water molecules.

**Figure 17:**
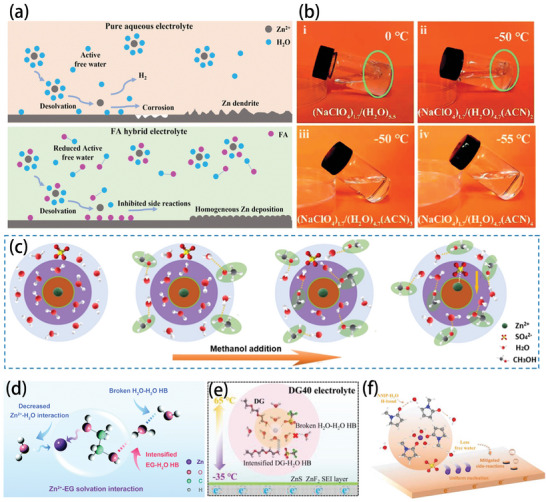
Schematic illustrations: a) Various reaction processes of the Zn^2+^ solvation shell, showing that the corresponding interfacial interaction between the pure aqueous electrolyte and the FA–H_2_O hybrid electrolyte would be beneficial. Reproduced with permission.^[^ ^103^ ^]^ Copyright 2023, RSC Publishing. b) The state of NaClO_4_‐based solutions with different H_2_O/ACN molar ratios at low temperatures. Reproduced with permission.^[^ ^3e^ ^]^ Copyright2020, Royal Society of Chemistry. c) The alteration in the Zn^2+^ solvation shell upon methanol introduction. Reproduced with permission.^[^ ^121^ ^]^ Copyright 2021, Wiley‐VCH GmbH. d) How Zn^2+^‐EG solvation interactions affect the chemistry of mixed electrolytes by possible mechanisms. Reproduced with permission.^[^ ^17b^ ^]^ Copyright 2022, RSC Publishing. e) The interfacial chemistry on a Zn electrode for DG40. Reproduced with permission.^[^ ^154^ ^]^ Copyright 2023, Wiley‐VCH GmbH. f) The solvation structure of Zn^2+^ and the corresponding deposition behavior in NMP5 electrolyte. Reproduced with permission.^[^ ^155^ ^]^ Copyright 2022, Wiley‐VCH GmbH.

In addition to destroying the HB network structure of water molecules, DMSO can also stabilize ions, by having higher absorption energy towards Zn^2+^ and the (002) plane. DMSO regulates the solvation structure of Zn^2+^ and induces Zn^2+^ to form a more stable form. It is an effective additive in ZnSO_4_ electrolytes for inhibiting side reactions and dendrites Through its influence on HBs in metal ions and water molecules, in symmetrical Zn||Zn batteries it can facilitate good performance at 20 °C and −20 °C. It can remain stable for more than 2100 hours under all conditions without producing dendrites and by‐products.^[^ ^119^ ^]^ FA, which also has high absorption energy toward (002) surfaces, can not only act as an HB acceptor and donor to regulate the HB network of water molecules in an aqueous solution, but also regulates the solvation structure of Zn^2+^ through strong coordination with it, replacing some water molecules in the solvation sheath and achieving uniform Zn deposition (**Figure** **17**a) while promoting excellent low‐temperature properties.^[^ ^103^ ^]^

Schemes for stable operation of aqueous electrolytes at low temperatures due to the high polarity and low viscosity of ACN have been reported. Sun et al. studied the mechanism of introducing organic solvents and found that ACN molecules are strongly coordinated with Na^+^, thus destroying the cation‐anion AGGs in the electrolyte. The solvation structure transformation shifts from aggregations towards CIPs and/or SSIP. This transformation weakens the interaction between anions and cations, effectively hindering the precipitation of NaClO_4_. The Na^+^ ion solvation structure also shows that ClO_4_^−^, H_2_O, and ACN could interact with Na^+^ ions in a mixed solution. The optimized NaClO_4_/H_2_O/ACN electrolyte remains a transparent solution at −50 °C (Figure 17b) with an ionic conductivity of 5.58 mS cm^−1^.^[^ ^3e^ ^]^ The ACN/water cosolvent electrolyte can then break the HB network to adjust the structure of the Zn^2+^ solvation sheath, which promotes ion‐pair dissociation and reduces the size of the Zn^2+^ solvation shell, and thus, it greatly facilitates the Zn^2+^ transport and alleviates the concentration polarization of Zn^2+^. As a result, stable cycling of Zn–Zn symmetric batteries for more than 500 h is achieved under challenging conditions: at 5 mA cm^−2^ under −40 °C or at 10 mA cm^−2^ under −20 °C, using an electrolyte composed of 1 m Zn(CF_3_SO_3_)_2_ in ACN/H_2_O (72 vol% ACN + 28 vol% H_2_O).^[^ ^120^ ^]^

In methanol‐based anti‐solvent electrolytes, water molecules interact with methanol, and the activity of free water is reduced, weakening the solvation effect of Zn^2+^ in the electrolyte in aqueous batteries. In an antisolvent electrolyte with 50% methanol by volume (Anti‐M‐50%), the reversible capacity of Zn was significantly improved even at −20 °C and 60 °C, with the average Coulombic efficiencies of Anti‐M‐50% being 98.7% and 98.1%, respectively. Raman spectroscopy shows that the electron density of ^2^H increases due to the formation of HBs between ^2^H in D_2_O and O in methanol. As the volume ratio of methanol increases, these complexes gradually insert themselves into the outer and inner sheaths of Zn^2+^ (Figure 17c), seriously affecting the solvation of Zn^2+^ and ultimately destroying the coordination balance of water and Zn^2+^ in the inner sheath.^[^ ^121^ ^]^ Due to the unique solvation interaction between EG and Zn^2+^, as shown in Figure 17d, the interaction between EG and H_2_O is strengthened, effectively disrupting the HB network within H_2_O molecules. This enhancement results in a lower freezing point of the mixed electrolyte, reaching −40 °C, and enhances its ionic conductivity. The introduction of EG partially reduces the solvation interaction between Zn^2+^ and H_2_O. It has been revealed that Zn^2+^ has strong solvation interaction with H_2_O and O atoms in EG molecules, which helps mitigate side reactions, such as the hydrogen evolution reaction on the Zn anode. The distinctive solvation interaction of Zn^2+^ with EG effectively strengthens the HBs between EG and H_2_O while reducing the solvation of Zn^2+^ with H_2_O, resulting in a hybrid electrolyte with a decreased freezing point and reversible Zn/Zn^2+^ chemistry.^[^ ^17b^ ^]^

Diethylene glycol monoethyl ether (DG) can also break the HBs between water molecules, forming a distinct PPS layer that involve both DG and OTF^−^ co‐participation, which disrupts the HB network and inhibits the formation of free water, and the reductive decomposition of DG and OTF^−^ can form a self‐healing ZnF_2_‐ZnS SEI, as shown in Figure 17e. This also effectively inhibits the occurrence of side reactions, so that a symmetric Zn battery containing it achieves long cycling lifespans of 1000 h at −35 °C.^[^ ^154^ ^]^

Liquid organic compounds containing carbonyl groups, such as N‐methyl pyrrolidone (NMP) and N,N‐dimethylformamide (DMF), are all highly polar solvents. These water‐soluble additives can act as proton acceptors to bond with dipole water molecules and destroy pre‐existing water clusters in aqueous solutions. The reversibility of Zn is achieved by introducing a carbonyl‐containing organic polar solvent into the Zn^2+^‐containing electrolyte. The solvation effect of water and NMP reduces the free water outside the solvation sheath. Representative electrolytes with NMP polar additive contribute to the structural reshaping of Zn^2+^ solvation and the stabilization of the HB network of water (Figure 17f).^[^ ^155^ ^]^ This is because the carbonyl functional group in NMP is an HB acceptor, which is different from the carbonyl‐containing molecules, DMF and dimethyl ketone (DMK), NMP has the largest solvent dipole moment (4.09) and a higher dielectric constant (33.0), which facilitates its insertion into the Zn^2+^ solvation sheath and the replacement of coordination H_2_O. Calculated ESP mapping shows that NMP has the highest electronegativity (‐5.135 eV), verifying that it has the strongest interaction with H_2_O/cations. The binding energy of the NMP‐H_2_O pair is ‐3.726 eV, which is lower than the −2.943 eV of the H_2_O‐H_2_O pair. This means that the original water HB network tends to reorganize, and more free water is captured and combined with polar NMP molecules.

#### 3.2.3 Weakening Ion–Ion Interactions

**Figure 18:**
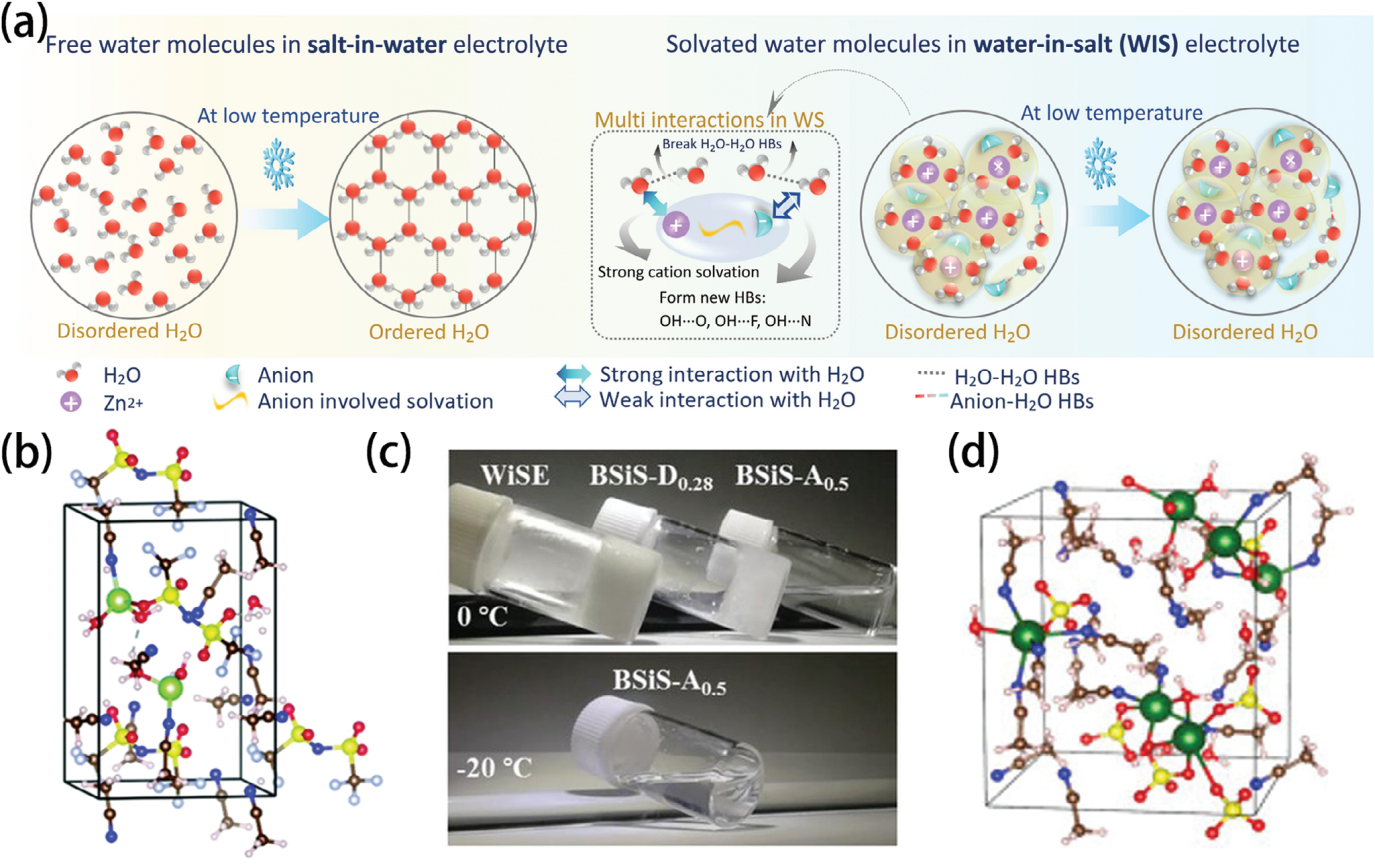
a) Schematic illustration of the interactions surrounding water molecules in salt‐in‐water and water‐in‐salt electrolytes under low‐temperature conditions. Reproduced with permission.^[^ ^135^ ^]^ Copyright 2022, The AAAS. b) 5 m AWIS electrolyte (LiTFSI(H_2_O)_2.6_(CH_3_CN)_3.7_) during DFT‐MD simulations. This diagram uses Li (green), O (red), H (pink), C (brown), N (blue), S (yellow), and F (gray) for atom representation. Reproduced with permission.^[^ ^158^ ^]^ Copyright 2018, RSC Publishing. c) Photographs showing the states of WiSE, BSiS‐D_0.28_, and BSiS‐A_0.5_ electrolytes at 0 and ‐20 °C. Reproduced with permission.^[^ ^160^ ^]^ Copyright 2019, WILEY‐VCH. d) DFT‐MD simulation of the NaClO_4_/(H_2_O)_1.5_(ACN)_2.4_ electrolyte, Na (green), O (red), H (pink), Cl (yellow), C (brown), and N (blue). Reproduced with permission.^[^ ^157a^ ^]^ Copyright 2019, Elsevier.

The fairly stable ultra‐concentrated WIS electrolytes have been successfully applied in battery systems such as aqueous Li‐ion batteries.^[^ ^16^, ^156^ ^]^ **Figure** **18**a shows free water molecules in a salt‐in‐water electrolyte and solvated water molecules in a salt‐in‐water electrolyte.^[^ ^135^ ^]^ The high salt/solvent molar ratio in the electrolyte will lead to low ionic conductivity and even salt out of the electrolyte at low temperatures, but the addition of a cosolvent in the high concentration electrolyte can inhibit the salt out at low temperatures, thereby improving the low temperature performance.^[^ ^3^, ^138^, ^157^ ^]^

Acetonitrile, an electrochemically inert organic solvent commonly used for solvation with its ‐C≡N functional group can act as an HB acceptor and electrostatically interact with OH‐ to break the HB network in water molecules. The high dielectric constant and low viscosity of ACN are also the reasons why it is the preferred organic solvent for optimizing WIS systems. Dou et al. developed a co‐solvent‐in‐salt approach by combining ACN with a standard WIS electrolyte (21 M LiTFSI/H_2_O) to create an “‘acetonitrile/water‐in‐salt”’ (AWIS) electrolyte. It was observed that the inclusion of ACN in the 5 M AWIS electrolyte created steric isolation, reducing the interaction forces between anions and cations. The ACN molecules could participate in the formation of the Li^+^ solvation shell, as presented in Figure 18b. This allowed a cell simulation of energy storage systems to work well over a wide temperature range from −30 to 50 °C.^[^ ^158^ ^]^ It happens that there is a similar case. Xiao and co‐workers recently introduced the concept of cosolvent systems in salt by mixing ACN with typical WIS electrolytes such as 21 m LiTFSI or 17 m sodium perchlorate (NaClO_4_). As a result of the strong coordination between water molecules and lithium ions, and the weakening of cation‐anion attraction after the addition of ACN, the AWIS electrolyte exhibited better conductivity, lower viscosity, and a lower freezing temperature while maintaining a broad electrochemical stability window (ESW).^[^ ^159^ ^]^ Chen et al. increased the LiTFSI salt concentration in AWIS to a super concentration (15.3 m) in this system, at which the formation of the interphase was activated. Due to the strong coordination between water molecules and Li^+^ and the reduced cation‐anion attraction following the addition of ACN, the ACN/ AWIS electrolyte demonstrated increased conductivity, decreased viscosity, and lower freezing points, all the while preserving a wide ESW. Due to the low freezing point of ACN, the electrolyte with the addition of acetonitrile remained liquid at ‐20 °C (Figure 18c).^[^ ^160^ ^]^ Dou et al. took the same aqueous‐organic hybrid electrolyte and came up with an optimal molar ratio of electrolyte salt‐water‐ACN of 1/1.5/2.4 (i.e., 8 m NaClO_4_), and the water molecules were entirely coordinated with Na^+^ cations, resulting in minimal hydrogen‐bond interaction (Figure 18d), which presented the optimal combination of ESW, conductivity, viscosity, and safety. All the water molecules are coordinated with Na^+^, and thus the AWIS showed a broad ESW of ≈3.16 V, while there were almost no free water molecules in the water. The WIS electrolyte significantly extended the voltage window of water batteries and supercapacitors, and the addition of ACN can effectively alleviate the problem of degradation or even failure of battery performance due to the precipitation of a high concentration of salt at low temperature.^[^ ^157a^ ^]^ The advantages of high conductivity, low viscosity, good wettability, and a wide ESW can also be achieved by adding ACN to a WIS electrolyte based on LiTFSI mixed electrolyte.^[^ ^159^ ^]^

Recently, significant progress has been made on solid‐state electrolytes (SSEs) to deliver desirable performance at low temperatures.^[^ ^161^ ^]^ Typically, SSEs can be divided into inorganic solid electrolytes (ISEs), solid polymer electrolytes (SPEs), and organic/inorganic composite electrolytes.^[^ ^162^ ^]^ Unlike organic liquid electrolytes solidify easily at sub‐zero temperatures, SSEs could remain solid over a wide temperature range without completely losing ionic conductivity.^[^ ^163^ ^]^ For instance, Li et al. designed a high‐entropy lithium superionic conductor analogy LSiGePSBrO with high entropy strategy by anion and cation substitution, which achieved a record‐breaking ionic conductivity 32 mS cm^−1^ at 25 °C and retained an ionic conductivity of ≈1 mS cm^−1^ even at −50 °C. Consequently, the all‐solid‐state cell with a high‐loading cathode (thickness 800 µm) incorporating LSiGePSBrO as the catholyte demonstrated a discharge capacity of 17.3 mAh cm^−2^ at −10 °C.^[^ ^164^ ^]^

For SPEs, crystalizing is an obstacle to high ionic conductivity at low temperatures. As such, composite electrolyte design by mixing organic and inorganic substances with inorganic fillers has been frequently reported. The combined electrolytes could reduce the crystallinity of organic solid electrolytes, and provide additional ion transfer channels to achieve high ionic conductivity as well as high mechanical properties of solid electrolytes.^[^ ^165^ ^]^ Lv et al. proposed that crosslinking can improve the mechanical strength of the composite and reduce the crystallinity of the organic solid electrolytes. An amorphous PEO‐based solid‐state composite electrolyte was reported by ultraviolet polymerizing PEO and methacryloyloxypropyltrimethoxy silane (KH570)‐modified SiO_2_ which demonstrates both satisfactory mechanical performance and high ionic conductivity at room (3.37 × 10^−4^ S cm^−1^) and low temperatures (1.73 × 10^−4^ S cm^−1^ at 0 °C). It is demonstrated that, in addition to reducing the crystallinity of PEO through crosslinking, a rapid Li^+^ ion transfer zone is generated. Moreover, kh570‐modified SiO_2_ inorganic particles can also promote the dissociation of lithium salt by Lewis acid center, which further improves the ionic conductivity of the composite electrolyte. The development of SSEs is still in the infancy stage, and the charge transfer mechanisms through the SSE structures are still unclear. Further explorations are required to accelerate the practical application of low‐temperature solid‐state batteries based on SSEs with high ionic conductivity.

## 4 Electrode Design

At low temperatures, the main reason for the deterioration of battery performance is the slow dynamics, in addition to the previously mentioned slow transfer of ions in the bulk electrolytes and in desolvation and internal ion transport at the SEI, but also includes the difficult diffusion of ions inside the electrodes.^[^ ^166^ ^]^ Although ion transport in the electrolyte and electrodes controls the rate at low temperatures, the electrode material itself will have problems such as capacity attenuation during the cycling, especially at low temperatures.^[^ ^84^, ^167^ ^]^ Therefore, this section will discuss the approaches to improve the low‐temperature performance of rechargeable batteries by shortening the transmission path and changing the spatial structure through reasonable structural engineering electrode design.

### 4.1 Anode

#### 4.1.1 Size Reduction

Size reduction is a commonly used method to mitigate the capacity attenuation and efficiency reduction caused by sluggish ion embedding and removal at low temperatures, which is effective for reducing the distance for the ion transfer. For example, nano‐alloys can increase the specific surface area of the electrode, providing more ion diffusion channels and active sites, which improves the slow kinetics at low temperatures.^[^ ^167^, ^168^ ^]^

**Figure 19:**
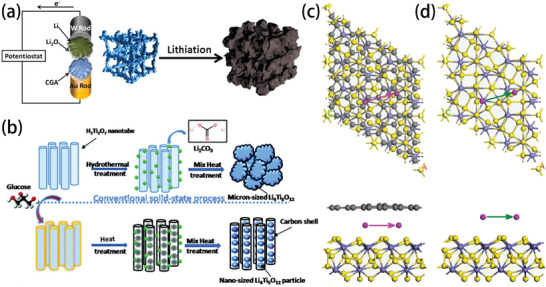
a) Schematic illustration of the cell configuration for the lithiation process in the CGA‐6 system. Reproduced with permission.^[^ ^170^ ^]^ Copyright 2019, American Chemical Society. b) Schematic presentation of the conventional process for the NSFLTOFC composite and the new strategy for the peapod‐like NSFLTOFC composite. Reproduced with permission.^[^ ^175^ ^]^ Copyright 2016, RSC Pub. c) Na atoms migrate under the FeS_2_/graphene heterointerface. d) The surface of single FeS_2_. Reproduced with permission.^[^ ^176^ ^]^ Copyright 2019, WILEY‐VCH.

Recently, an Al‐based nanoporous alloy anode has been developed for Li metal batteries. The nano‐pore structure of Cu–Ge–Al ternary alloy (NP‐CuGeAl) prepared by selective etching of aluminum exhibits an interconnected network with pores and ligaments. The three‐dimensional bi‐continuous and layered porous structure and a variety of intermetallic compounds (M*_x_* N*_y_*, M, N = Cu, Al, Ge) could enhance the rate capability and cycling performance, and also adapt to the volume change of the alloy‐type anode simultaneously.^[^ ^169^ ^]^ At −20 °C, the performance of CGA‐48 (etched for 48 h) was significantly better than that of CGA‐6 (**Figure** **19**a).^[^ ^170^ ^]^ These pores formed by acid etching provide good interstitial spaces for volume regulation during discharge and also provide good channels for electrolyte penetration and rapid kinetics.^[^ ^171^ ^]^

Numerous reports have demonstrated that size reduction can be achieved by introducing functional carbon into the electrode material, which can also improve the electronic conductivity of the electrode. It was found that the charge transfer resistance of metal materials increased significantly with decreasing temperature, while that for carbon‐based materials was not significantly affected, rendering carbon material modification as a feasible approach to the development of low‐temperature batteries.^[^ ^172^ ^]^ It has been frequently reported that carbon nanotubes (CNTs) can be used as composite materials with nanoalloys because they could provide a transport network for electrons and reduce the diffusion resistance inside the electrode.^[^ ^173^ ^]^ For instance, when nanoscale iron particles were introduced into single‐walled carbon nanotubes (SWNTs), the resulting Fe@SWNT anode provided an initial Coulombic efficiency of 53.1%, which was much higher than that for the pure SWNTs (35.6%). When the current density was 150 mA g^−1^, Fe@SWNTs provided a reversible capacity of ≈283 mAh g^−1^ at 5 °C and 142 mAh g^−1^ at −15 °C, which is much better than ≈172 mAh g^−1^ at 47 mAh g^−1^ for pure SWNT anodes, respectively.^[^ ^174^ ^]^ Similarly, a composite anode with carbon fiber/tube‐coated peapod‐like nano‐sized Li_4_Ti_5_O_12_ (NS‐LTO‐C) has been reported for LIBs (Figure 19b), in which the nanoparticles can greatly accelerate the charge and discharge speed of lithium ions and shorten the charge transfer paths between the active material and the electrolyte.^[^ ^175^ ^]^ The improvement in electrochemical performance is mainly due to the synergistic effect of the uniform carbon coating with excellent electronic conductivity, large specific surface area, and nanometer size of the active material, and the unique structure of the pod‐like NS‐LTO‐C composite.

Carbon nanotechnology has also been applied to sodium‐ion batteries (SIBs). Chen et al. reported a FeS_2_@graphene@carbon nanofiber (FeS_2_@G@CNF) anode for SIBs. The morphology characterization showed that graphene could not only effectively reduce the size distribution of FeS_2_ nanoparticles in the nanofibers, but also enhance the degree of graphitization and the electrical conductivity of the composite. Na^+^ migrates more easily at the FeS_2_/graphene heterointerface than at the single FeS_2_ surface (Figure 19c,d). The highly reversible electrochemical reaction between FeS_2_@G@CNF and Na^+^ also benefited from the highly conductive fiber network and the good protective effect of graphene‐coated FeS_2_ nanoparticles. At −20 °C, the discharge capacity of the full cell (FeS_2_||Na_2_V_3_(PO_4_)_3_) could also reach 43 mAh g^−1^.^[^ ^176^ ^]^

In short, when the electrode size is reduced to the nanometer level, the increase of the specific surface area inside the electrode increases the number of ion channels and active sites, and greatly improves the diffusion speed of internal ions. Therefore, the reduction of particle size can significantly improve the fast charging capability.^[^ ^167^, ^177^ ^]^ In addition, nanoscale anodes could promote rapid charging by increasing the contact area between the electrode and the electrolyte, thereby reducing ion transport diffusion lengths. The combination with carbon nanoparticles once again provides new transmission paths. Furthermore, the reduced diffusion length and increased surface area to volume ratio can effectively mitigate the decrease in conductivity at low temperatures.^[^ ^178^ ^]^ However, it is worth noting that a high specific surface area means high surface energy, which will increase the instability of the electrode to a certain extent, such as with the self‐aggregation of some nanoparticles, so nanosize is one of the parameters that need to be carefully controlled in nanoelectrode technology.

#### 4.1.2 Spatial Structure Engineering

Carbon‐based anodes have been widely used in various rechargeable batteries,^[^ ^179^ ^]^ being the primary choice of anode material for a wide variety of rechargeable battery systems.^[^ ^180^ ^]^ For instance, graphite anodes have been widely used in LIBs. The diffusion of ions in graphite is the main factor that determines the anode dynamics. However, the slow diffusion of ions can also aggravate lithium plating.^[^ ^9^, ^35^, ^181^ ^]^ The slow kinetics caused by the long ion diffusion distance has been a notorious issue. The Li^+^ in a graphite anode has a diffusion coefficient of only about 10^−11^ cm^2^ s^−1^ in the direction of the transmission plane, and it diffuses slowly at sub‐zero temperatures, even resulting in the plating of Li metal on the electrode surface. To shorten the ion transport distance of Li^+^ through changing the spatial structure and thus accelerating the ion transport dynamics has been proved to be an effective method to improve low temperature performance.^[^ ^109^, ^182^ ^]^

**Figure 20:**
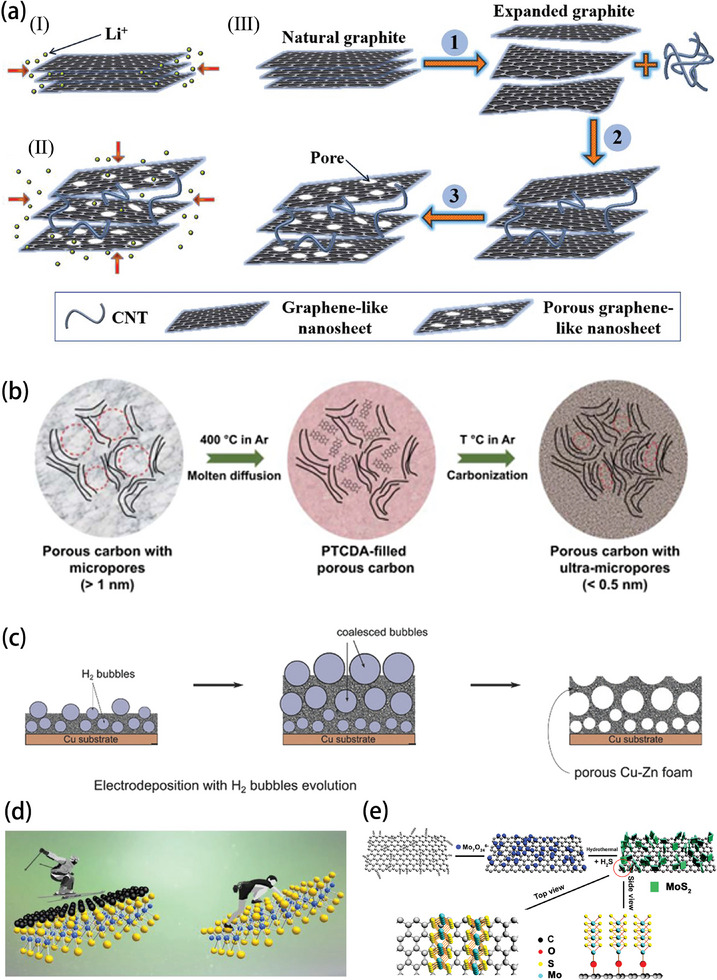
a) Schematic illustration of Li^+^ insertion process for I) natural graphite and II) porous graphite nanosheet (PGN). III) The preparation process for PGN/CNT. Reproduced with permission.^[^ ^184^ ^]^ Copyright 2019, Elsevier. b) The operating principle of the dynamic hydrogen bubble template DHBT method.^[^ ^185^ ^]^ c) Melt diffusion‐carbonization strategy scheme (where T represents the carbonization temperature). Reproduced with permission.^[^ ^186^ ^]^ Copyright 2017, WILEY‐VCH. d) Schematic illustration of ionic diffusion in MoS_2_/C and pure MoS_2_. Reproduced with permission.^[^ ^187^ ^]^ Copyright 2020, Elsevier. e) Schematic diagram of the MoS_2_/G synthesis process. Reproduced with permission.^[^ ^188^ ^]^ Copyright 2016, American Chemical Society.

Graphene holds great promise in nanoelectronics and energy storage/conversion, composites, and other applications. From a practical application point of view, 2D graphene has proven to be a promising alternative to graphite anode materials due to its unique structure, excellent electrical and mechanical properties, high surface‐to‐volume ratio, and chemical stability.^[^ ^183^ ^]^ To further shorten the diffusion path, Xu et al. developed a favorable electrode consisting of porous graphite nanosheets (PGN) with through‐holes and carbon nanotubes (CNTs) (**Figure** **20**a). The through holes effectively reduce the diffusion path length, while the CNTs effectively inhibit the aggregation of porous graphene nanosheets (PGN), mitigating the tendency of two‐dimensional nanoparticles to restack. When combined with a low‐dissolution energy electrolyte that can run smoothly at −20 °C, the LIBs delivered excellent rate performance at room temperature with a long cycle life of >500 cycles at 90% capacity retention, even at 4 C‐rate cycles.^[^ ^184^ ^]^ It was found that the main factor limiting the embedding of lithium ions in PGN/CNT was the charge transfer resistance at the liquid‐solid interface, rather than the diffusion in PGN/CNT particles. This dissolving process prevents solvent molecules around Li^+^ ions (i.e., solvation) from entering the electrode, especially at low temperatures.^[^ ^39^ ^]^

The formation of pores in carbon‐based materials is also a strategy for expanding the transmission path and providing outstanding rate performance by altering the spatial structure. The insertion barrier caused by the large radius of sodium ions can also be alleviated by the intervention of the pores.^[^ ^189^ ^]^ Yang et al. reported that the electrochemical performance of electrodes can be improved through introducing micropores, specifically, a carbon material constructed by the diffusion of molten aromatics into microporous carbon and then carbonization, which can effectively increase the micropores inside the carbon material (Figure 20b). These micropores allow only bare Na^+^ to enter in the absence of electrolyte solvents, which effectively reduces the decomposition of electrolytes and provides additional sodium ion storage sites, increasing the electrode capacity, with a capacity of 5.32 mAh cm^−2^ at −20 °C.^[^ ^185^ ^]^ Non‐carbon‐based zinc‐rich porous Cu–Zn alloys also exhibited excellent low temperature properties due to the 3D interconnected cavernous structure formed by macroscopic pores. The stable output of ≈200 mAh g^−1^ at −20 °C was because the presence of pores is particularly suitable for the migration of Li^+^ from solution to porous electrode materials (Figure 20c).^[^ ^186^ ^]^

2D MoS_2_ is a prototypical transition metal sulfide exhibiting a layered structure like graphite. It consists of a single‐atom‐thick Mo sheet sandwiched between two layers of sulfur, forming a structure that is three atoms thick.^[^ ^188^ ^]^ It has good ion insertion/removal capability in a specific direction and has fast discharge/charge performance.^[^ ^190^ ^]^ Its conductivity is poor, however, and the structural deterioration in the process of battery charging and discharging will lead to decreased performance and capacity attenuation. In the composite anode design, the incorporation of MoS_2_ and carbon to form MoS_2_/carbon (MoS_2_/C) composites can combine the excellent electrical conductivity of carbon and the rapid charge and discharge capability of MoS_2_, which helps to improve the low‐temperature performance. The insertion of carbon between MoS_2_ layers leads to the expansion of the layer space, further enhancing the ion diffusion capacity, resulting in better rate and cycling performance.^[^ ^191^ ^]^ Liu et al. prepared MoS_2_/C hybrid thin layers, with capacities of 854.3 and 497.5 mAh g^−1^ at 100 and 1000 mA g^−1^ current densities at −20 °C, respectively. The carbon component in the compound can greatly promote the transfer of Li^+^ and electrons at low temperatures (Figure 20d).^[^ ^187^, ^192^ ^]^ When the edge Mo of the MoS_2_ nanosheets is directly coupled with the functional group oxygen (Mo/O/C bond) on the graphene, tMoS_2_ nanosheets are formed that are vertically connected to the graphene (Figure 20e). This not only effectively prevents the stacking of graphene sheets but also prevents the accumulation of MoS_2_ during the charging and discharging process. The interfacial interaction of the C/O/Mo bonds formed by MoS_2_/G can improve the electron transfer rate and structural stability of the MoS_2_/G electrode. In addition, the graphene sheet not only improves the electrical conductivity of the composite, but also acts as a substrate for uniformly dispersing the active MoS_2_ nanosheets, and it can also act as a buffer to adapt to changes in volume during the cycling.^[^ ^188^ ^]^ The long stable cycling brought about by the structural adjustment has brought enlightenment to the study of the structure at low temperatures.^[^ ^193^ ^]^

#### 4.1.3 Element Doping

**Figure 21:**
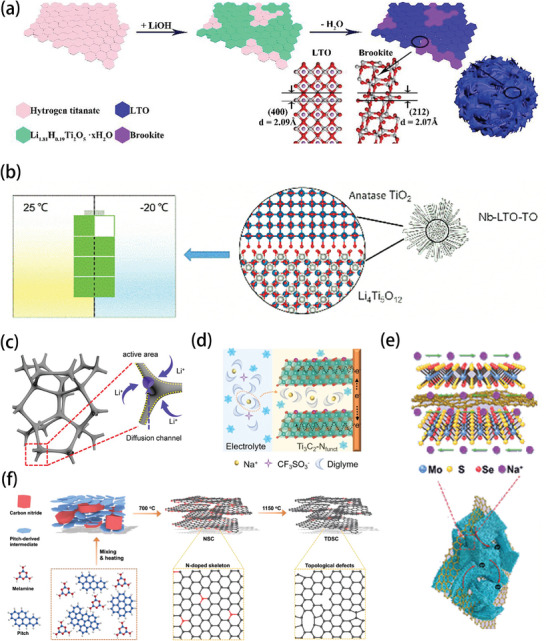
a) The mechanism for the formation of hierarchical porous LTO/brookite with an integrated structure. Reproduced with permission.^[^ ^197^ ^]^ Copyright 2019, Elsevier. b) Composite structure of Nb‐LTO‐TO and discharge at 1 C under −20 °C and normal temperature. Reproduced with permission.^[^ ^198^ ^]^ Copyright 2021, Published by Elsevier. c) The proposed lithium storage pathway for BNG tubular foam. Reproduced with permission.^[^ ^200^ ^]^ Copyright 2020, American Chemical Society. d) Illustration of the Na^+^‐solvent co‐intercalation behavior in Ti_3_C_2_‐N_funct_ at low‐temperature.^[^ ^202^ ^]^ e) Self‐assembling earthworm MoSSe nanoribbon. Reproduced with permission.^[^ ^203^ ^]^ Copyright 2020, Wiley‐VCH GmbH. f) Schematic diagram of the temperature‐programmed co‐pyrolysis strategy for TDSC synthesis. Reproduced with permission.^[^ ^204^ ^]^ Copyright 2023, Wiley‐VCH GmbH.

Element doping has been developed as one of the most effective approaches to regulate the microstructure and properties of electrode materials.^[^ ^194^ ^]^ For instance, Mg^2+^ and W^6+^ are suitable dopants to replace the Li^+^ and Ti^4+^ ions in the Li_2_ZnTi_3_O_8_ (LZTO) structure (**Figure** **21**a). Doping higher‐oxidation‐state cations into Li^+^ or Ti^4+^ sites can yield higher intrinsic conductivity because the additional charge can be compensated by lattice defects or an increase in electron concentration. Doping could improve the ionic conductivity of LZTO and stabilize the crystal structure. At 0 °C, the 60th cycle still yielded 192.9 mAh g^−1^ at 0.6 A g^−1^, with no capacity attenuation at 200 and 300 cycles at 0.2 and 0.5 A g^−1^, respectively.^[^ ^195^ ^]^ Lv et al. introduced the concept of ion fill coefficient, which leads to a much stable space and ion transport channel for electrode. With the introduction of Ca^2+^ the ion fill coefficient, the electrode has the Mg_1.5_Ca_0.5_Nb_34_O_87_ delivers a 1.6 times faster Li^+^ diffusivity at −20 °C, leading to 56% higher reversible capacity and 1.5 times higher rate capability than Mg_2_Nb_34_O_87_.^[^ ^194^ ^]^ W‐doped LTO/brookite layered porous spheres composed of thin nanosheets have been reported for LIBs, which could not only provide abundant phase boundaries and a large number of defects, as well as reducing the barrier against charge transport, but also shorten the ion transport path, so as to achieve excellent performance at low temperature for fast charging in LIBs.^[^ ^196^ ^]^ Moreover, the layered porous configuration of LTO/brookite enhances the contact surface area between the electrode material and the electrolyte, facilitating increased availability of lithium insertion sites and thereby enhancing electrochemical performance. As a result, it delivered a high specific capacity of up to 195 mAh g^−1^ at −20 °C.^[^ ^197^ ^]^ In Nb‐doped Li_4_Ti_5_O_12_‐TiO_2_ hierarchical microspheres, doping with Nb^5+^ partially reduced Ti^4+^ to Ti^3+^, improved the electrical conductivity of the composite, enlarged the lattice space, and increased the diffusion coefficient of Li^+^. Compared with undoped samples, the Li^+^ diffusion coefficient of the niobium‐doped sample increased from 1.969 × 10^−14^ to 1.004 × 10^−12^ cm^2^ s^−1^, which improved the low‐temperature performance of the electrode, and the discharge capacity of 119.4 mAh g^−1^ could be reached at −20 °C, even at a high current rate of 5 C, and reached 128.6 mAh g^−1^ under a current rate of 1 C at −20 °C, which represents 85.8% utilization of the capacity of Nb‐LTO‐TO, where TO is TiO_2_, at room temperature (Figure 21b).^[^ ^198^ ^]^

Non‐metal element doping has also been reported to improve the low temperature performance of the electrodes. For instance, N atoms can be easily doped into the TiO_2_ structure to promote the impurity conductivity of TiO_2_, and N‐doped TiO_2_/TiN/graphene nanocomposites were prepared by using incompletely oxidized TiN (TiO_2_@TiN) as an intermediate, which improved the ionic and electrical conductivity of TiO_2_ at low temperatures. The binding of graphene also improves electron transfer on the TiO_2_ surface by connecting loose one‐dimensional TiO_2_ nanotubes. Even at 5 A g^−1^ (≈15 C) at −20 °C, the electrodes still showed a specific capacity of 74 mAh g^−1^.^[^ ^178e^ ^]^ Lu et al. reported the preparation of branched N‐doped graphitic tubular foam (BNG) by the chemical vapor deposition (CVD) process. The introduction of nitrogen atoms could expand the interlayer spacing between the graphite layers and further modify the electronic structure at the curved junction, which is not only conducive to the transmission of Li, but also in which the active polar *sp* ^3^ hybrid C‐N group can optimize the storage performance of Li^+^ compared with the original non‐polar *sp* ^2^‐C network. The C─N group that dominates the pyridine/pyridine absence forms curved junctions and expanded planar branches in the BNG tubular foam (Figure 21c), resulting in significantly improved insertion/de‐insertion and diffusion kinetics of Li^+^ at low temperatures.^[^ ^199^ ^]^ At −10 °C, the specific capacity of BNG tubular foam reached 222.5 mAh g^−1^ after 150 cycles at 0.1 C, which was 1.75 times higher than that of commercial multi‐wall carbon nanotubes (MWCNT).^[^ ^200^ ^]^

Elemental doping is also used in sodium batteries. Composite anodes coated with ultrafine ZnSe nanoparticles in nitrogen‐doped porous carbon nanofiber composites (ZnSe@NCNFs) have been reported for SIB applications, showing excellent electrochemical properties. The results demonstrated that the porous structure and ultrafine ZnSe nanoparticles as well as the strong N‐doped carbon matrix could collectively establish a permeable network that facilitates rapid ion and electron transfer. The unique carbon nanofibers effectively inhibit the agglomeration of ZnSe nanoparticles, which could maintain stable structure with a one‐dimensional carbon framework and also could shorten the Na^+^ diffusion distance and accelerate electron transfer. The assembled SIBs delivered stable cycling at the low temperatures of −20 to −40 °C.^[^ ^201^ ^]^ Yang et al. proposed an interlayer restriction strategy to customize the nitrogen end (Ti_3_C_2_‐N_funct_) on Ti_3_C_2_ MXene to address these issues. The introduction of a nitrogen terminal enables Ti_3_C_2_‐N_funct_ to have a larger interlayer space and adjust the in‐plane structure of Ti_3_C_2_, which not only improves the electrical conductivity, but also provides enough Na^+^ adsorption sites, further improving the possibility of Ti_3_C_2_ being able to accommodate more Na atoms and enhancing the Na^+^ storage capacity of Ti_3_C_2_. In addition, it also shows Na^+^‐solvent co‐intercalation behavior (Figure 21d), which can effectively avoid the high solvent removal energy barrier at low temperatures. The capacity retention rate reached 80.9% at −25 °C after 5000 cycles.^[^ ^202^ ^]^

For MoS_2_, in addition to recombination with carbon to adjust the layer spacing and optimize the electrochemical performance, recent studies have noted that the metal conductivity and weak van der Waals interaction obtained in 1T‐MoS_2_ can also have a positive effect.^[^ ^205^ ^]^ When Se^2−^ is introduced into MoS_2_, a 2H/1T hybrid phase is formed (Figure 21e), which increases the layer spacing of MoS_2_, and the newly synthesized MoSSe has both the high energy density of MoS_2_ and the excellent cycling stability of MoSe_2_. When graphene oxide (GO) nanosheets were added to the system, the MoSSe nanorods were uniformly attached to the graphene oxide nanosheets due to the induction of the GO functional groups, forming a three‐dimensional conductive network and maintaining sufficient space between the nanorods. The unique morphology of MoSSe@rGO improved ion/electron transfer kinetics and structural stability, resulting in a capacity of 533.9 mAh g^−1^ at 0 °C, and a capacity retention of 87.8% compared with room temperature.^[^ ^203^ ^]^

It has been reported that defect engineering can also achieve the effect of enlarging the layer spacing as does the doping of elements. Yang et al. introduced a straightforward programmed temperature co‐pyrolysis method to efficiently synthesize topologically defective soft carbon (TDSC) using inexpensive bitumen and melamine (Figure 21f). This synthesis method optimizes the skeleton structure of TDSC, shortens the graphite‐like microcrystals, widens the interlayer spacing, and adds rich topological defects (such as pentagonal, heptagonal, and octagonal defects), which enhance the adsorption capacity of the carbon framework for K^+^, thus significantly reducing the resistance of K^+^ to embedding deep in the layer. Under these two superposition effects, TDSC exhibited fast pseudocapacitive K^+^ intercalation behavior and could maintain capacity of 144 mAh g^−1^ at −10 °C, showing great potential at low temperatures.^[^ ^204^ ^]^

### 4.2 Cathode

Like the anode, the cathode of a rechargeable battery also experiences degradation at low temperatures. The capacity decay of cathode materials at low temperatures is mainly caused by three factors: 1) increased lattice instability of the cathode materials, resulting in the capacity decline; 2) increased electrochemical impedance and charge transfer resistance, resulting in reduced electrochemical reactions; and 3) the limited diffusion of ions within the crystal and the reduced ionic diffusion coefficient. Compared with the anode materials at low‐temperature, cathode materials have been less studied. Recent studies have revealed that size reduction, functional coating, and element doping are favorable strategies to enhance the low temperature performance of rechargeable batteries.

#### 4.2.1 Size Reduction

**Figure 22:**
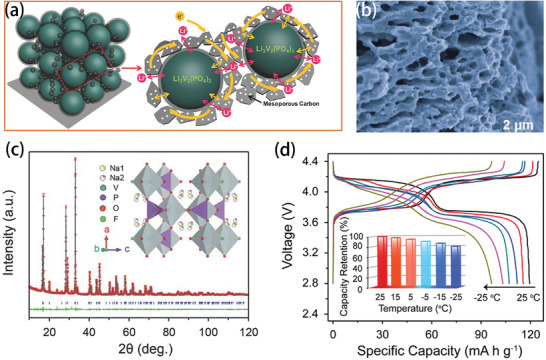
a) Illustration of the electron and Li^+^ channels of layered carbon‐modified Li_3_V_2_(PO_4_)_3_. Reproduced with permission.^[^ ^208^ ^]^ Copyright 2014, WILEY‐VCH. b) Scanning electron microscope (SEM) images of the NVP/C‐F. Reproduced with permission.^[^ ^211^ ^]^ Copyright 2020, Wiley‐VCH GmbH. c) Structural and morphology characterizations of NVPF‐NTP with its powder X‐ray diffraction (PXRD) pattern and Le Bail refinements for NVPF‐NTP. Reproduced with permission.^[^ ^209^ ^]^ Copyright 2017, WILEY‐VCH. d) The galvanostatic charge‐discharge (GCD) curves and capacity retention of MCMB||NVPOF hybrid Li/Na‐ion batteries (HLNIBs) cycled at 0.013 A g^−1^ in the temperature range from 25 to −25 °C. Reproduced with permission.^[^ ^210^ ^]^ Copyright 2018, WILEY‐VCH.

The reduction of the cathode particle size can effectively improve its kinetics and low‐temperature performance because small particles can shorten the diffusion paths of electrons and ions, and provide a larger specific surface area for ion insertion/extraction.^[^ ^206^ ^]^ For instance, it has been reported that monodisperse V_2_O_5_ nanostructures can be used in the form of arrays of V_2_O_5_ nanorods with a diameter of 70 nm to form Li cathode materials. Due to the reduced size, the Li^+^ diffusion distance is shortened, and the performance at −20 °C is enhanced.^[^ ^207^ ^]^ When the coated carbon layer is on the nanoscale (**Figure** **22**a), the cathode can also have good temperature adaptability. The specific discharge capacities can reach 106 mAh g^−1^ at 5 C and −20 °C.^[^ ^208^ ^]^ For instance, Na_3_V_2_(PO_4_)_2_O_2_F nano‐tetra prisms (NVPOF‐NTP) have been reported as cathode materials for SIBs (Figure 22b), which exhibit excellent rate capabilities as a result of their nanoscale particle size and their high ion diffusion coefficient due to the large surface area. During Na insertion/extraction, the volume change of the NVPOF lattice was as low as 2.56%, with a capacity of 96.1 mAh g^−1^, equivalent to 0.2 C and −25 °C.^[^ ^209^ ^]^ The NVPOF cathode showed good low‐temperature performance in the full cells with mesophase carbon micro beads as anode, delivering a capacity of 96.4 mAh g^−1^ at −25 °C (Figure 22c).^[^ ^210^ ^]^ It was also reported that carbon‐based nanocrystals of NVP could also accelerate the ion transport dynamics, so its 3D porous foam structure was used to further accelerate the dynamics (Figure 22d). A full battery composed of an NVP/C‐F cathode could provide a reversible capacity of 105 mAh g^−1^ at 0.2 C and −20 °C.^[^ ^211^ ^]^

#### 4.2.2 Functional Coatings

Recent studies have revealed that surface coating is one of the most effective ways to alleviate the capacity decay of rechargeable batteries at low temperatures.^[^ ^87^, ^193^, ^212^ ^]^ At present, surface coating as a heterogeneous material on particles of the electrode materials with a thickness of several nanometers has been demonstrated to be an effective approach to enhance the low temperature performance of electrodes.^[^ ^213^ ^]^ The coating on the particles usually plays a role in stabilizing the structure of the cathode material to prevent the side reactions with the electrolyte, while the cathode surface could also enhance the stability of the cathode, as well as improving the electrical conductivity and ionic conductivity.^[^ ^212^, ^214^ ^]^ Different coatings are being explored by more researchers because they can achieve higher performance and better low temperature effects.^[^ ^206b,c^ ^]^ Coating the particles of the cathode is easy to conduct and has been applied to a variety of cathode materials. Recently, AlF_3_‐coated Li_1.2_Ni_0.13_Co_0.13_Mn_0.54_O_2_ was prepared via a facile wet chemical process by Zhao and colleagues. The results demonstrated that, on the surface of the cathode particles, there exists an optimal performance via a dense, nanoscale‐thick AlF_3_ coating, which not only establishes a fast Li^+^ transport bridge for adjacent active materials, enhancing the diffusion rate of Li^+^, but also reduces side reactions between the electrolyte and the active materials. Compared with the original cathode, the 2% AlF_3_ coated samples showed significantly better rate capability and higher capacity retention at −20 °C.^[^ ^215^ ^]^

**Figure 23:**
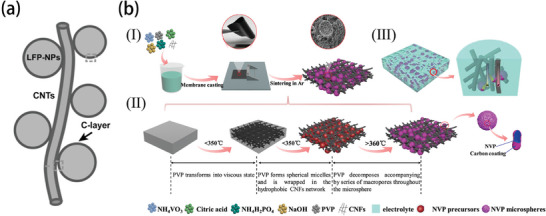
a) Schematic illustration of the preparation of the LFP@C/CNT nanocomposites. Reproduced with permission.^[^ ^216^ ^]^ Copyright 2013, WILEY‐VCH. b, I) Synthetic process, II) formation mechanism of porous NVP microspheres wrapped in interwoven CNF networks, and III) electron and ion transport mechanisms in the electrode. Reproduced with permission.^[^ ^220^ ^]^ Copyright 2020, American Chemical Society.

Carbon coating of LFP is also a common method to solve low‐temperature problems. Recently, a modified LFP cathode decorated with amorphous carbon coatings and graphitized conductive carbon has been reported for LIBs, which delivered a high capacity retention rate of 71.4% at ‐25 °C. The enhanced cycling performance at low temperatures is attributed to the synergistic effects of the amorphous carbon coating and graphitized conductive CNTs. The carbon coating on LFP nanoparticles facilitates Li^+^ diffusion and stabilizes the interface, while the CNTs electronically connect all the LFP@C particles, forming an efficient three‐dimensional conductive network across the electrode (**Figure** **23**a), which reduces the internal resistance, thereby further improving the electrochemical performance of LFP.^[^ ^216^ ^]^ In another study, carbon coating and phosphorus doping (LFP/C‐P) have been developed for LFP cathode, which could protect the surface of the electrode from corrosion by the electrolyte, and reduce the barrier against Li^+^ diffusion during Li^+^ intercalation. At the low temperature of −40 °C, the initial specific discharge capacity at 0.1 C reached 82.7 mAh g^−1^.^[^ ^217^ ^]^ Given the ease with which these structures or similar structures can be manufactured, this type of technology also gives inspiration for the rational design of other electrode materials. Functional coatings can not only accelerate the ion diffusion rate but also stabilize the structure, which is favorable for long‐term cycling at low temperatures. For instance, Sun et al. reported a greener diazonium soft‐chemistry method to graft a conductive polymer coating on the surfaces of LiNi_0.6_Co_0.2_Mn_0.2_O_2_ (LNCM‐3) particles with a particle size of 3 µm, which delivered outstanding low‐temperature performance as cathode in LIBs.^[^ ^218^ ^]^ The results demonstrated that the LNCM‐3 particles were uniformly encased in a thin polystyrene film by the spontaneous reaction of C_6_H_5_N_2_ and BF_4_^−^. Compared with the uncoated LNCM‐3, the low‐temperature discharge capacity, discharge rate, and low‐temperature long‐term cycling stability of polyphenylene/LNCM‐3 were significantly improved.^[^ ^219^ ^]^

Similarly, coating technology is also widely used in sodium batteries.^[^ ^221^ ^]^ A discontinuous charge transfer network generated by NVP@C microspheres in synergy with CNFs could achieve facile ion diffusion, in which the interwoven CNFs are employed as efficient electron transport highways. The high efficiency of this dual continuous independent electrode provides a new idea for low‐temperature batteries (Figure 23b).^[^ ^220^ ^]^ Similarly, a sodium super ionic conductor (NASICON)‐type NaTi_2_(PO_4_)_3_ nano‐shell coating on P2‐type Na_0.6_7Co_0.2_Mn_0.8_O_2_ (NCM) surfaces has been reported for SIBs, in which the optimized coating can effectively inhibit the side reactions and the dissolution of Mn^3+^, greatly improving the cycling stability and improving the kinetic and structural stability of NCM. The performance of the coated cathode delivered a high discharge capacity of 120.9 mAh g^−1^ at −20 °C and 0.2 C.^[^ ^222^ ^]^ It has also been reported that, when nanoscale NVP or NaTi_2_(PO_4_)_3_ is embedded into the carbon skeletons to form a three‐dimensional layered porous structure, not only can the nanoparticles accelerate the diffusion kinetics, but also the carbon skeletons can accelerate the electron conduction and stabilize the strain caused by Na^+^ (de)intercalation. The C‐F||NVP full‐cell can provide a reversible capacity of 105 mAh g^−1^ at 0.2 C and −20 °C.^[^ ^211^ ^]^

#### 4.2.3 Element Doping

**Figure 24:**
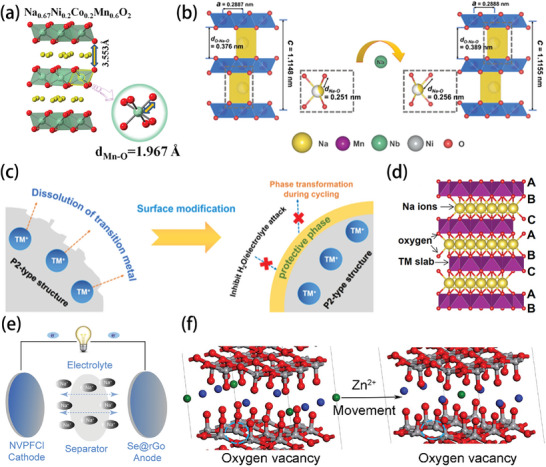
a) Illustration of the effect of Co^3+^ doping on electrode structure. Reproduced with permission.^[^ ^224^ ^]^ Copyright 2021, Elsevier. b) Effect of Nb doping on the crystal structure. c) Schematic illustration of the protective effect on the bulk structure.^[^ ^225^ ^]^ d) The crystal structure of O3‐type NaNi_0.5_Mn_0.5_O_2_ (O3‐NaNM). Reproduced with permission.^[^ ^223b^ ^]^ Copyright 2022, American Chemical Society. e) Illustration of Se@rGO||NVPFCl full batteries assembled with NVPFCl cathode and Se@rGO anode. Reproduced with permission.^[^ ^227^ ^]^ Copyright 2021, Elsevier. f) Schematic illustration of Zn^2+^ diffusion in NH_4_V_4_O_10‐_ *_x_* with oxygen vacancy at the terminal site. Reproduced with permission.^[^ ^228^ ^]^ Copyright 2020, Elsevier.

Element doping has also been widely used for cathode materials to enhance the low temperature performance.^[^ ^223^ ^]^ It has been reported that, in an SIB with Na_0.67_Ni_0.33_Mn_0.67_O_2_ as cathode with Na^+^/vacancy order (**Figure** **24**a), the Jahn‐Teller octahedral distortion of Mn^3+^ can be solved by suitable Co‐substituted Na_0.67_Ni_0.2_Co_0.2_Mn_0.6_O_2_ microspheres. The results demonstrated that the introduction of Co can increase the layer spacing, which results in small fluctuations in the diffusion coefficient of Na^+^, even at −40 °C. At −40 °C, a high reversible discharge capacity of 132.2 mAh g^−1^ at 0.2 C was achieved, representing 80% of the capacity observed at room temperature.^[^ ^224^ ^]^ Similarly, Nb substitution for Ni can regulate the bond length of transition metal (TM)‐O and Na–O, promoting high mobility and a low activation energy barrier for Na^+^. Additionally, Nb‐doping induces surface preformation that facilitates the formation of a stable CEI film. This film helps to prevent phase transitions and surface degradation, thereby stabilizing the (de)intercalation of Na^+^ ions during cycling, as presented in Figure 24b,c.^[^ ^225^ ^]^ The Sb substitution is effective in extending layer spacing (Figure 24d) and suppressing the irreversible P′3‐O3′ phase transition, as a result, the Sb‐doped O_3_‐NaNi_0.5_Mn_0.5_‐_x_Sb_x_O_2_ cathode has little capacity loss after hundreds of cycles at temperatures as low as −20 °C.^[^ ^223b^ ^]^ It has also been reported that metal ions introduced into the V_2_O_5_ interlayer can increase interlayer spacing and increase ion diffusion ability.^[^ ^226^ ^]^

Apart from doping with metal elements, a recent report demonstrated that Cl atoms can be incorporated into the NVPOF lattice to deliver a 3D open channel framework.^[^ ^227^ ^]^ The introduction of F/Cl atoms into the NASICON lattice can generate high ionicity and strong induction effects between adjacent V atoms, which is helpful for enhanced structural stability of the Na_3_V_2_(PO_4_)_2_O_2_F_0.99_Cl_0.01_ (NCPFCl) cathode. As a result, the full cells with the NVPFCl cathode and Se@rGO anode delivered stable cycling over a wide temperature range from −25 to 50 °C (Figure 24e).^[^ ^227^ ^]^ It has also been reported that vacancies can also be introduced into the cathode lattice to enhance the low‐temperature performance. The controlled introduction of defects into the electrode can also improve the electrode conductivity and provide ion diffusion channels, thus overcoming migration and diffusion barriers.^[^ ^229^ ^]^ For example, oxygen‐deficient NH_4_V_4_O_10‐_ *_x_* ·*n*H_2_O microspheres have been reported as cathode materials for zinc ion batteries, and oxygen vacancy in the lattice could reduce the Zn^2+^ diffusion energy barrier (Figure 24f), which enables the rapid diffusion of Zn^2+^ over a wide temperature range to deliver good electrochemical performance. Consequently, such a cell delivered a high capacity of 296 Wh kg^−1^ at ‐30 °C.^[^ ^228^ ^]^ A metal oxide with excessive defects may lead to sudden degradation or even collapse of the structure, however, which will threaten the reversible capacity and cycle life of the battery. The trade‐off between high mobility and structural stability has to be considered. This prompted work to continue to conduct visualization studies and characterization methods to better evaluate changes in batteries.^[^ ^230^ ^]^

**Figure 25:**
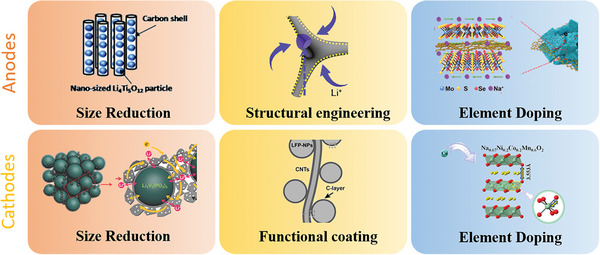
Low‐temperature optimization strategies for anodes and cathodes.

The approaches to enhance the low temperature performance of the rechargeable batteries via electrode material modifications can be summarized as in **Figure** **25**. The key issue is to enhance the internal ion transport speed in the electrode materials. In the size reduction strategy, the sites and channels for ion transport increase due to the specific surface area caused by decreasing electrode particle size. This undoubtedly allows more ions to undergo electrochemical reactions simultaneously, thereby mitigating slow kinetics at low temperatures. Spatial structure optimization is applied to the anode based on carbon‐based materials, and a composite of different materials can not only solve the agglomeration problem of two‐dimensional structures such as graphene or MoS_2_, but also further improve the quality of the transmission channel. Element doping also expands ion transport channels through introducing heteroatoms to increase phase boundaries and defects. This results in an increase in ion transport channels within the electrode. Additionally, doping higher oxidation state cations can enhance the intrinsic conductivity of the anode. The introduction of vacancies can achieve similar effects as element doping, although attention must be paid to controlling macrostructure stability. Coating on cathodes can also partially reduce barriers for ion transmission. Overall, these strategies contribute significantly towards enhancing the low‐temperature performance of the electrodes and therefore the rechargeable batteries.

## 5 Conclusions

**Figure 26:**
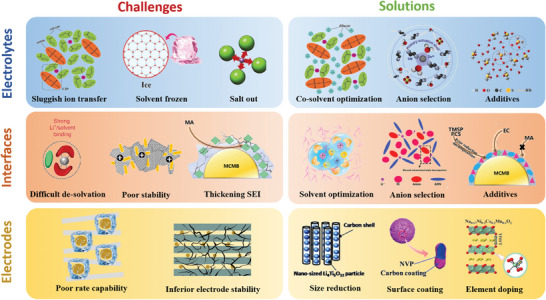
Challenges and solutions for low‐temperature rechargeable batteries.

In summary, the low temperature performance of rechargeable batteries is essentially important for their practical application in daily life and beyond, while challenges remain for the stable cycling of rechargeable batteries in low temperatures. Deterioration of the electrolytes, electrodes, and interfaces are all responsible for the deterioration of their electrochemical performance in low temperatures. In this review, the remaining challenges, failure mechanisms, and feasible strategies for aqueous and non‐aqueous rechargeable batteries working at low temperatures have been discussed in detail (**Figure** **26**). The ion transfer kinetics in the electrolyte, interfaces, and the electrodes have been elucidated from an atomic scale perspective. At present, some achievements on low‐temperature rechargeable batteries have been made, but there are still many challenges to be solved.

1. The working mechanism and the interactions of various species in the aqueous and non‐aqueous electrolytes have been intensively discussed since the properties of the electrolyte determine the low‐temperature performance of the rechargeable batteries. One of the major concerns for the inferior low‐temperature performance of rechargeable batteries is the low ionic conductivity of the electrolyte, for which the electrolyte solvents are essentially important. The desolvation process at the interfaces determines the electrochemical performance of the batteries, and the de‐solvation energy is related to the solvent–solvent, ion–solvent, and ion–ion interactions in the solvation structure of the charge carriers. The de‐solvation energy of the cations can be reduced by introducing electrolyte solvents with low dielectric constants, while this may lead to reduced ionic conductivity of the electrolyte due to lower dissolvability of the electrolyte salts in low dielectric solvents. Functional groups, including B, S, P, and F, can be introduced into the solvents to lower the HOMO and alter the solvation structure, among which F‐containing solvents or additives have been frequently utilized in non‐aqueous electrolytes to produce salt‐rich stable interfaces. Despite the advantages of F‐containing solvents, there are some overlooked drawbacks should be aware of. Fluorinated solvents are generally more expensive; and the impact of the fluorinated solvents on the manufactural instruments and the environmental issues related to fluorine release from spent batteries should be carefully evaluated. The fundamental working mechanisms of the functional groups, such as the influence of the number and position of the atoms in the solvent molecules, should be further explored. For aqueous batteries, anti‐solvents are frequently used to reduce the activity of the water molecules. The breakdown of the HBs network can reduce the freezing point of the electrolyte but increase of the HBs strength is conducive to extending the electrochemical window of the electrolyte. It is worth noting that the electrolyte salts for aqueous electrolytes are more important than for non‐aqueous electrolytes. The dissociation of the electrolyte salts in aqueous electrolytes would generate a solution with various pH values, which is critical for the stability of the electrodes and the interfaces. Briefly, the key for the electrolyte design of low‐temperature rechargeable batteries is to balance the interactions of various species in the solution, the ultimate preference is a mixed solvent with low viscosity, low freezing point, high salt solubility, and low desolvation barrier.
2. The interfaces on the electrodes are vitally important for the cycling stability of the batteries. The formation of the interfaces is directly influenced by the electrolyte, for which the solvation structure of the cations plays a crucial role in determining the structure and components. The solvation structures with AGGs and CIPs could facilitate the generation of inorganic salt‐rich interfaces with enhanced structural stability, while the contact of cations and anions in these structures is prone to cause salt out at low temperatures. Up to date, the exact constitutions of the interfaces and the roles of the various components are still unclear. Various kinds of crystalline inorganic species have been identified in the interfaces, while it is challenging to determine the amorphous organic species. The migration pathway of the ions through the interfaces with complicated components is still ambiguous. Besides, the dynamic evolutions of the interfaces at varied temperatures and prolonged cycling times have rarely been understood, and controllable modifications of the thickness and composition of the interfaces are still challenging to realize.
3. The low‐temperature performance of the electrodes is largely determined by the interfaces on the electrodes and the inherent structure of the active materials. Size reduction of the electrode material is a straightforward approach to enhance the low‐temperature performance, which could physically decrease the length of the ion transportation. Besides, slow ion and electron transfer rates in the structure of the solid electrode material may also hinder the reaction equilibrium and the available capacity of the electrode. Element doping has been utilized to increase phase boundaries and defects within the electrodes to enhance the intrinsic conductivity and stabilize the interfaces, while excessive doping has adverse effects on the structural stability of the electrode materials.
4. To achieve a deeper understanding of the working mechanism of the batteries at operating conditions, novel in‐situ and operando characterizations are required. For instance, the exact composition of the interfaces and the formation mechanisms relating to the solvation structure of the cations, the desolvation of the ions and migration through the interfaces, and the dynamic evolution of the interfaces need to be studied by various in operando characterizations. At the microscale, in‐situ optical techniques and in situ Raman analysis could also be utilized to observe the morphology evolution of the electrodes and solvation structure of the electrolytes. At the nanoscale, in‐situ SEM and TEM techniques have been developed for batteries with liquid electrolytes, although the resolution may be hampered due to the disruption of the electron beams by the thin‐layer liquid electrolyte.^[^ ^231^ ^]^ Synchrotron‐based in‐operando characterizations with high intensity and deep penetration depth have been frequently utilized to study the working mechanism of rechargeable batteries in a non‐destructive manner, such as X‐ray diffraction (XRD),^[^ ^232^ ^]^ X‐ray pair distribution function (XPDF),^[^ ^233^ ^]^ and X‐ray absorption (XAS).^[^ ^234^ ^]^
5. Theoretical studies, such as DFT calculations, have been frequently utilized to determine the HOMO and LUMO of the electrolyte solvents and guide the electrolyte design, while the calculations for complicated solvation structures under the influence of the interfaces are challenging. Other than conventional labor and time‐consuming academic research, machine learning could establish models to describe non‐explicit relations between the input parameters and the output results, by combining various properties of the battery components from known databases and collecting experimental data from different reports. This approach could compensate for the drawbacks of traditional theoretical calculations based on a few hundred molecules yet lacking of experimental supports, it can also be used to predict new phenomenon and discover novel materials that have never been reported.

It is anticipated that the low‐temperature performance of the rechargeable batteries can be further improved with the emerging innovations in electrolyte engineering, interface optimization, electrode design, *in operando* characterizations, and machine learning studies. Rechargeable batteries with desirable electrochemical performance at low temperatures would be indispensable for human activities in extreme climates to power a sustainable world.

## Conflict of Interest

The authors declare no conflict of interest.
